# Influence of *ATXN2* intermediate CAG repeats, 9bp duplication and alternative splicing on SCA3 pathogenesis

**DOI:** 10.1186/s40478-025-02074-0

**Published:** 2025-07-19

**Authors:** Marilena Lauerer, Jennifer Faber, Nicolas Casadei, Magda M. Santana, Georg Auburger, Michaela Pogoda, Jakob Admard, Lea Kaupp, Patricia Laura Kos, Mafalda Raposo, Manuela Lima, Luis Pereira de Almeida, Hector Garcia-Moreno, Paola Giunti, Jeroen de Vries, Bart P. van de Warrenburg, Judith van Gaalen, Marcus Grobe-Einsler, Berkan Koyak, Kathrin Reetz, Friedrich Erdlenbruch, Heike Jacobi, Jon Infante, Holger Hengel, Ludger Schöls, Thomas Klockgether, Olaf Rieß, Jeannette Hübener-Schmid, ESMI study group

**Affiliations:** 1 Institute for Medical Genetics and Applied Genomics, University of Tübingen, Nägelestraße 5, 72074 Tübingen, Germany; 2 Center for Rare Disease, University of Tübingen, Tübingen, Germany; 3 German Center for Neurodegenerative Diseases, Bonn, Germany; 4 Center for Neurology, Department of Parkinson’s Disease, Sleep and Movement Disorders, University Hospital Bonn, University of Bonn, Bonn, Germany; 5 Department of Neuroradiology, University Hospital Bonn, Bonn, Germany; 6 NGS Competence Center Tübingen, Tübingen, Germany; 7 Center for Neuroscience and Cell Biology (CNC), University of Coimbra, Coimbra, Portugal; 8 Experimental Neurology, Clinic of Neurology, Medical School, Goethe University, Frankfurt am Main, Germany; 9 IBMC – Instituto de Biologia Molecular e Celular, Instituto de Investigação e Inovação em Saúde, Universidade do Porto, Porto, i3S Portugal; 10 UMIB - Unit for Multidisciplinary Research in Biomedicine, ICBAS - School of Medicine and Biomedical Sciences, University of Porto, Porto, Portugal; 11 Faculdade de Ciências e Tecnologia, Universidade dos Açores, Ponta Delgada, Portugal; 12 Center for Innovative Biomedicine and Biotechnology (CIBB), University of Coimbra, Coimbra, Portugal; 13 Institute for Interdisciplinary Research, University of Coimbra (IIIUC), Coimbra, Portugal; 14 Faculty of Pharmacy, University of Coimbra (FFUC), Coimbra, Portugal; 15 Ataxia Centre, Department of Clinical and Movement Neurosciences, UCL Queen Square Institute of Neurology, University College London, London, UK; 16 Department of Neurogenetics, National Hospital for Neurology and Neurosurgery, University College London Hospitals NHS Foundation Trust, London, UK; 17 University Medical Center Groningen, Neurology, Groningen, The Netherlands; 18 Department of Neurology, Donders Institute for Brain, Cognition, and Behaviour, Radboud University Medical Center, Nijmegen, The Netherlands; 19 Department of Neurology, Rijnstate Hospital, Arnhem, The Netherlands; 20 Department of Neurology, RWTH Aachen University, Aachen, Germany; 21 JARA-BRAIN Institute Molecular Neuroscience and Neuroimaging, Research Centre Juelich GmbH and RWTH Aachen University, Aachen, Germany; 22 Department of Neurology and Center for Translational Neuro- and Behavioral Sciences (C-TNBS), University Hospital Essen, University of Duisburg-Essen, Essen, Germany; 23 Department of Neurology, University Hospital of Heidelberg, Heidelberg, Germany; 24 University Hospital Marqués de Valdecilla-IDIVAL, Santander, Spain; 25 Centro de investigación biomédica en red de enfermedades neurodegenerativas (CIBERNED), Universidad de Cantabria, Santander, Spain; 26 Department of Neurodegenerative Diseases, Hertie Institute for Clinical Brain Research & Center of Neurology, University of Tübingen, Tübingen, Germany

**Keywords:** *ATXN2*, Gene modifier, Genotype-phenotype correlation, MJD, SCA3

## Abstract

Spinocerebellar ataxia type 3 (SCA3) is a neurodegenerative disease whose exact disease pathogenesis is not yet fully understood. We performed a genetic in-depth analysis of *ataxin-2* (*ATXN2)*, a gene that has already been described as a modulator of neurodegenerative diseases. We focused on the influence of an intermediate CAG repeat, a 9bp duplication (9bp), and isoform expression of *ATXN2* on the pathogenesis of SCA3.

Clinical and genetic data from a large European SCA3 cohort (total 390 probands) were analyzed. Fragment analyses were performed to determine the cytosine-adenine-guanine (CAG) repeat length and the 9bp duplication in *ATXN2*. RNA sequencing was performed on blood and cerebellum to evaluate *ATXN2* isoform profile. Cell culture and SCA3 mice were used to investigate the influence of intermediate ATXN2 length on ataxin-3 protein abundance, aggregation, and cell viability.

SCA3 carriers with an intermediate *ATXN2* repeat presented a significant increase in non-ataxic symptoms. A greater age at onset and faster disease progression were found in SCA3 carriers with a 9bp duplication. Co-expression of ATXN2 and ATXN3 in cell models revealed an influence of ATXN2 on ataxin-3 abundance and aggregation patterns. Determination of soluble ATXN2 abundance demonstrated a significant genotype-independent reduction in mouse brain. Aggregate analyses indicated that ataxin-2 is not co-localized with ataxin-3-containing aggregates.

Our comprehensive genetic study confirmed *ATXN2* as a modulator of SCA3 pathogenesis, including onset and presence of clinical symptoms. For the first time, the ATXN2 isoform profile was compared in blood and cerebellar tissue, revealing a unique profile depending on the genotype and tissue. Here, a significant higher expression of ATXN2 splice variant type I in blood and significantly lower expression in cerebellar tissue were found compared to ATXN2 splice variant type II. Molecular and biochemical analyses in SCA3 mice and cell culture provide further evidence on mechanistic aspects, including differences in protein abundance and co-aggregation propensity. In summary, our study provides new insights into the modulatory effects of *ATXN2* on SCA3 pathogenesis.

**Graphical Abstract:**

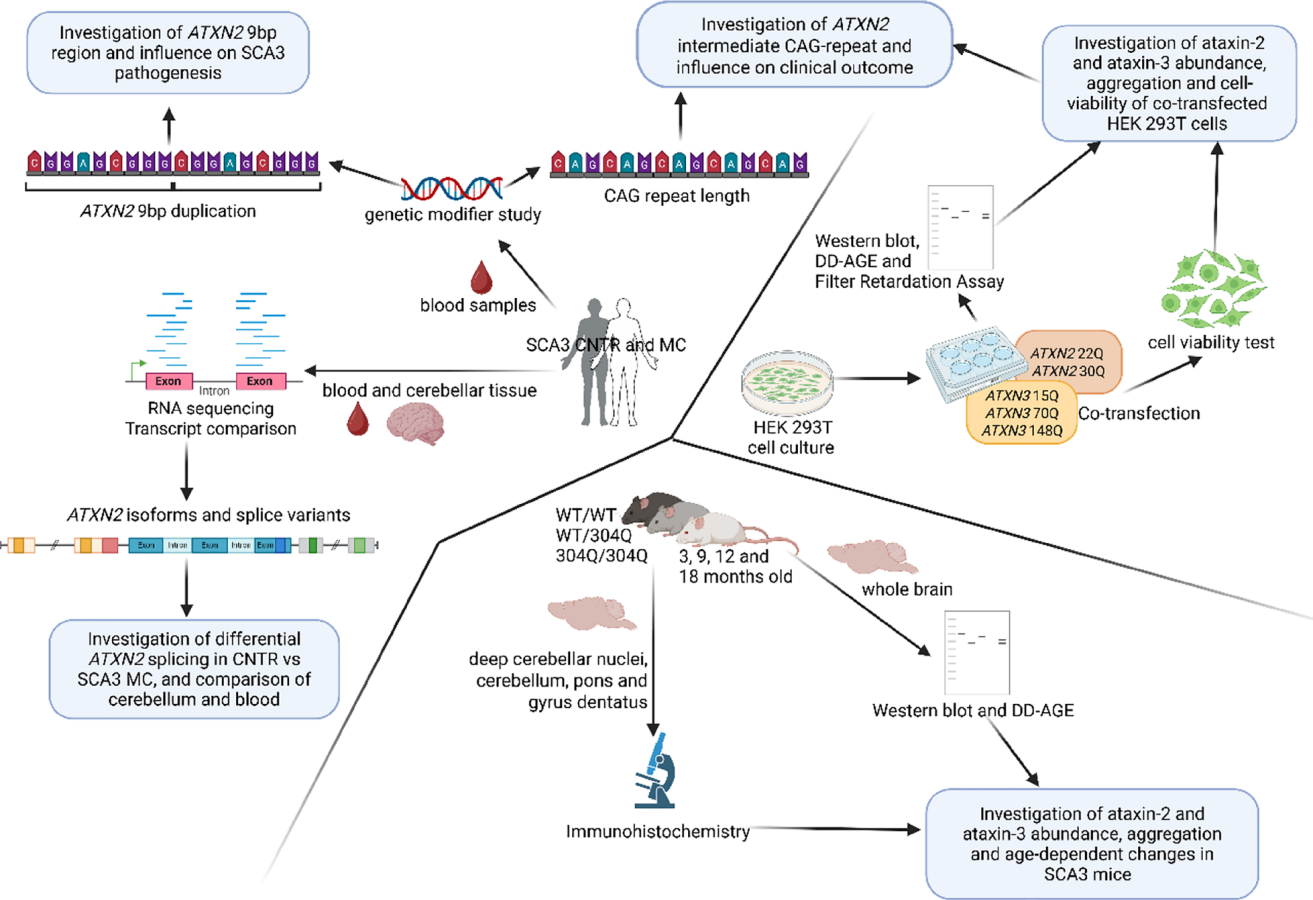

**Supplementary Information:**

The online version contains supplementary material available at 10.1186/s40478-025-02074-0.

## Introduction

Spinocerebellar ataxia type 3 (SCA3), also known as Machado-Joseph disease (MJD), is one of seven SCAs caused by cytosine-adenine-guanine (CAG) expansion in total of eleven known polyglutamine (polyQ) diseases [1–5]. With approximately 1:50,000-100,000 affected people, SCA3 is believed to be the most frequent autosomal ataxia [2, 6]. The disease-causing mutation in SCA3 is an expanded CAG repeat in the *Ataxin-3* gene (*ATXN3*) encoding a prolonged glutamine stretch in the ataxin*-*3 protein, leading to toxic accumulation in neurons [1, 7]. The CAG repeat length in chromosomes of healthy individuals varies from approximately 11–44, whereas in affected individuals, the expanded repeat length ranges from 47 to 87 repeats [6–12]. This CAG repeat length directly correlates with the severity of the clinical presentation and inversely correlates with the age at onset (AAO) of SCA3 [2, 10]. Frequent symptoms, among others, are progressive ataxia, spasticity and ophthalmoplegia [1, 13, 14]. Currently, no curative therapy is available for SCA3; therefore, a better understanding of the disease itself and the development of diagnostic tools are important for future clinical studies [1, 7].

In addition to CAG repeat length as a disease-modifying factor, there are other poorly understood mechanisms and genetic modifiers that contribute to high variability in the clinical presentation and rate of decline in SCA3 [6]. For example, the CAG expansion in *ATXN3* was found to explain only approximately 50–80% of the variance in AAO [15–17]. One formerly described modifier is the cytoplasmatic localized polyglutamine-repeat protein ATXN2, which plays an important role in RNA metabolism and translation regulation [18–22]. *ATXN2* is the causative gene for SCA2 when it contains an expanded CAG repeat of ≥ 33 [7, 23]. In healthy individuals, the CAG repeat sequence of *ATXN2* can vary from < 22 (short), 22 (medium), 23–26 (intermediate short) to 27–33 (intermediate) repeats, whereas 90% of the normal population harbors 22 repeats [7, 20, 24–27]. In particular, *ATXN2*, with an intermediate CAG repeat length, was previously described as a modifying factor for an earlier AAO in amyotrophic lateral sclerosis (ALS) patients, ATXN2 is associated with autosomal-dominant Parkinson’s disease and has been described as a potential modifying factor for an earlier onset in SCA3 patients [7, 17, 28–31]. A more recent study revealed a very rare 9bp (bp) duplication in the *ATXN2* promotor/exon 1 region [32]. Duplications were identified only in a few probands and only in *ATXN2* alleles with normal CAG repeat lengths [32]. Although only a few cases were identified, one study suggested an effect of this 9bp duplication (c.109-117delinsCGGAGCGGG) and demonstrated an earlier AAO of SCA3 and ALS [32]. Forty-three transcripts (splice variants) of *ATXN2* (GRCh38) were described by Ensembl.com [33]. In addition, further splice variants of *ATXN2* were previously described in the literature but are not necessarily the same as the splice variants listed by Ensembl.

In the present study, we analyzed the potential influence of *ATXN2* on SCA3 pathogenesis in one of the largest cohorts in which *ATXN2* has been analyzed in relation to SCA3 (a total of 390 probands). Therefore, we focused on intermediate CAG repeats, the 9bp duplication and correlations with clinical data. For the first time, *ATXN2* isoform expression was evaluated in SCA3 disease context, and two different biomaterials, blood as a non-pathogenic biomaterial and post-mortem cerebellum biosamples as the primary pathogenesis area, were compared. To understand the molecular background of the intermediate ATXN2 repeat, cell culture experiments were performed.

## Materials and methods

### Study cohort and data collection

All participants were enrolled in the European Spinocerebellar Ataxia Type 3/Machado-Joseph disease initiative (ESMI). ESMI is a European multicenter prospective observational study with highly standardized biosampling and clinical assessment [34]; the study protocol is available online (https://ataxia-esmi.eu/study-protocols). The samples from SCA3 mutation carriers (MC) (*n* = 276) and healthy controls (CNTR) (*n* = 114), without neurological or psychiatric disease, were collected at centers in Coimbra (Portugal), Azores (Portugal), Santander (Spain), Groningen (Netherlands), Nijmegen (Netherlands), London (United Kingdom), Bonn, Heidelberg, Aachen, Essen and Tübingen (all Germany). The Scale for the Assessment and Rating of Ataxia (SARA) was used to determine the presence and severity of ataxia [35]. According to this SARA sum score, the mutation carriers were divided into a preataxic group (SARA < 3) and an ataxic group (SARA ≥ 3). The Inventory of Non-Ataxia Signs (INAS) was used to assess neurological symptoms other than ataxia. It results in 16 subcategories, including hyperreflexia, areflexia, extensor plantar, spasticity, paresis, muscle atrophy, fasciculation, myoclonus, rigidity, chorea/dyskinesia, dystonia, resting tremor, sensory symptoms, urinary dysfunction, cognitive dysfunction and brainstem oculomotor signs, which constitute the basis for a sum of non-ataxic symptoms [36]. The AAO of ataxic symptoms was defined as the reported onset of gait disturbances. For the determination of individual disease progression, cross-sectional disease progression (CSDP) was calculated by dividing the SARA sum by the disease duration (DD).

The number of subjects included in each analysis varied because not all clinical parameters were available for every individual. Therefore, the specific number of subjects analyzed is shown in each graph.

The study was approved by all local institutional review boards of all the participating centers and the respective ethics committees. Written informed consent was obtained from all study participants before enrollment.

### Determination of *ATXN2* CAG repeat length and *ATXN2* 9bp duplication via fragment length analyses

DNA extracted from EDTA blood samples was used for DNA analysis of *ATXN2* CAG repeat length and *ATXN2* 9bp (wildtype (wt) or duplication (dup)) region. Standard PCR was performed with 2 µl of 20 µM forward and reverse primers (Metabion International AG, Planegg, DE) (Table 1), 50 ng/µl DNA, 2.5 µl of 10x PCR-buffer (Qiagen, Hilden, DE), 0.5 µl of 40 mM dNTPs (Qiagen) and 0.2 µl of 5 units/µl Taq DNA Polymerase (Qiagen). The PCR for the CAG repeat also contained 0.5 µl of 25 mM MgCl_2_ (Qiagen) and 1.25 µl of DMSO (Sigma-Aldrich, St. Louis, USA), whereas for the 9bp PCR, 4 µl of 5x Q-Solution (Qiagen) was added. The remaining volume was filled with H_2_O Ampuwa (Fresenius Kabi, Bad Homburg, DE) to a total volume of 25 µl. The thermal cycling conditions were 95 °C for 5 min; 35 cycles of 30 s at 95 °C, 30 s at 59 °C for CAG primer annealing, and 67 °C for 9bp primer annealing, and 30 s of 72 °C; and a final elongation at 72 °C for 7 min. PCR products were analyzed with the fragment analysis program using the CEQ™ 8000 Genetic Analysis System Sequencer (Beckman Coulter Inc., Brea, USA).

**Table 1:** Utilized primers of CAG repeat and 9bp region fragment length analysis

	primers	Sequence (5’-3’)	Product length[bp]	Annealing Temperature [°C]
CAG repeat ATXN2	forward, 5’ IRD700 fluorescently labeled	GGG CCC CTC ACC ATG TCG CTG	130 (*23 CAG repeats)*	68
	reverse	CGG GCT TGC GGA CAT TGG CAG		67
9bp region ATXN2	forward, 5’Cy5 fluorescently labeled	GCT GAA GGA ATA CAG TAG GAG AAG A	556	60
	reverse	CCG TTG CTA CCA AAA CAG TCT G		60

### RNA sequencing of human post-mortem brain and blood samples

#### Sample Preparation

RNA sequencing of human post-mortem brain material was performed earlier and is described in [37]. PAXgene blood RNA tubes were centrifuged for 10 min at 5000 × g and the resulting pellet was resuspended in a proteinase K mixture and inserted into a Qiasymphony (Qiagen, Hilden, Germany). Elution was performed using 80 µl of elution buffer, as provided in the QIAsymphony PAXgene Blood RNA Kit. The RNA concentration was measured via a Qubit RNA BR or HS Assay Kit (ThermoFisher Scientific, Waltham, USA). The RNA integrity number (RIN) was determined via the Fragment Analyzer 5300 and the Fragment Analyzer RNA kit or, in some cases, the BioAnalyzer2100 and the RNA 6000 Pico or the RNA 6000 Nano Kit (all Agilent Technologies, Santa Clara, USA).

### RNA sequencing

For library preparation of RNA isolated from blood, the mRNA fraction of 500 ng of total RNA was enriched via the NEBNext Poly(A) mRNA Magnetic Isolation Module (New England Biolabs, Ipswich, USA) for polyA capture. Next, libraries were prepared via the NEBNext Ultra II Directional RNA Library Prep Kit for Illumina and the NEBNext Multiplex Oligos for Illumina (both from New England Biolabs), according to the manufacturer’s instructions, automated on a Biomek i7 (Beckman Coulter, Brea, USA). Library molarity was determined by measuring the library size via the Fragment Analyzer 5300 and the Fragment Analyzer DNA HS NGS fragment kit (Agilent Technologies, Santa Clara, USA) and by measuring the library concentration via the Qubit Fluorometric Quantitation and dsDNA High Sensitivity Assay (Thermo Fisher Scientific, Waltham, USA). The libraries were denatured according to the manufacturer’s instructions, diluted and sequenced as paired-end 100 bp reads on an Illumina NovaSeq 6000 (Illumina, San Diego, USA) with a sequencing depth > 45 million clusters per sample.

The read quality of the RNA-sequencing data in fastq files was assessed via QoRTs to identify sequencing cycles with low average quality, adaptor contamination, or repetitive sequences from PCR amplification. Reads were aligned using STAR allowing gapped alignments to account for splicing against a custom-built genome composed of the Ensembl.org *Homo sapiens* GRCh38 [33], and alignment quality was analyzed via samtools and visually inspected via the Integrative Genome Viewer. Normalized read counts for all genes were obtained using Subread and edgeR. ´.

The RNA sequencing methods used for post-mortem brain samples have already been described elsewhere [37].

For the sake of simplicity, we refer to all transcript variants published by Ensembl as *ATXN2* isoforms, whereas specific transcript variants with isolated exon variation are referred to as splice variants (types I-VI, corresponding to the literature).

### Cell culture and transfection

The cultivation of HEK293T cells (ATCC: CRL-3216) was performed at 37 °C and 5% CO_2_ in Dulbecco’s Modified Eagle Medium (DMEM; Gibco, Thermo Fisher Scientific) supplemented with 10% fetal bovine serum (Roche Diagnostics GmbH, Mannheim, DE) and 1% antibiotic-antimycotic mixture (Gibco, Thermo Fisher Scientific). The cells were passaged twice a week at 90% confluence.

GFP-tagged plasmids encoding *ATXN3* with different polyQ repeat lengths (pEGFP-N2-Ataxin3-15Q, pEGFP-N2-Ataxin3-70Q and pEGFP-N2-Ataxin3-148Q) were transiently transfected. Additionally, co-transfection was performed by combining pEGFP-N2-*ATXN3* constructs with HA-tagged *ATXN2* with different polyQ repeat lengths (pcDNA-*ATXN2*-22Q and pcDNA-*ATXN2*-30Q). These *ATXN2* plasmids were kindly provided by Nicolas Charlet Berguerand of IGBMC - Institut de Génétique et de Biologie Moléculaire et Cellulaire. HEK293T cells were transfected with the transfection reagent Attractene (Qiagen) according to the corresponding standard transfection protocol. Therefore, 400 000 cells were seeded 24 h before transfection into a six-well culture plate in DMEM, and transfection was performed with 1.2 µg of total plasmid DNA. Next, the cells were incubated for 72 h at 37 °C and 5% CO_2_. HEK293T cells were harvested with cold DPBS (Thermo Fisher Scientific) and centrifuged at 350 × g for 5 min to form a cell pellet after an additional washing step with DPBS. The cells were subsequently lysed in RIPA buffer (50 mM Tris, pH 7.45; 150 mM NaCl; 0.1% (w/v) SDS; 0.5% (w/v) sodium deoxycholate; 1% (v/v) Triton X-100) containing cOmplete™ protease inhibitor cocktail (Merck KGaA, Darmstadt, D) for 30 min on ice and vortexed every 10 min. Lysates for western blot analyses were obtained from homogenates (used for aggregate analyses) via a centrifugation step for 30 min at 4 °C and 13 200 × g. Last, the protein concentrations of the lysates and homogenates were determined spectrophotometrically via the Bradford reagent (Bio-Rad Laboratories).

### Cell viability test using presto blue

The viability of the transiently transfected HEK293T cells with the *ATXN3* and *ATXN2* constructs described earlier was tested according to the PrestoBlue™ Cell Viability Reagent manual (2019) in a 96-well plate. The test was performed over a period of 140 min, and the results of the transfections were compared statistically after an incubation time of 80 min (logarithmic phase).

### Western blot

Protein lysates for western blot analyses (*n* = 3) were prepared to a final protein concentration of 30 µg and therefore mixed with 25% (v/v) 4x LDS-buffer (2.5 M Tris pH 8.5; 50% glycerol; 2.5% phenol red; 2.1 mM EDTA; 294 mM LDS) and 100 mM DTT. Next, the samples were denatured in a thermoshaker at 70 °C and 600 rpm for 10 min.

Western blotting was performed following standard procedures described elsewhere [38]. Briefly, protein samples were separated electrophoretically via SDS-PAGE with the use of the Mini-PROTEAN^®^ Tetra Cell System (Bio-Rad Laboratories, Hercules, CA, USA). A 6% (w/v) acrylamide/bis-acrylamide gel was used as the stacking gel, and a 10% acrylamide/bis-acrylamide gel was used for sample separation. SDS-PAGE was performed at 100 V and 250 mA with 1x MOPS running buffer (50 mM MOPS, 50 mM Tris, 0.1% (w/v) SDS, 1 mM EDTA). A 0.2 μm pore size nitrocellulose membrane (Amersham Protran Premium, Cytiva, Marlborough, MA, USA) was used for protein transfer. This wet transfer was carried out at 80 V and 250 mA for 2 h in 1X Bicine/Bis-Tris transfer buffer (25 mM Bicine, 25 mM Bis-Tris, 1 mM EDTA) with 15% (v/v) methanol. After transfer, the membrane was blocked for 1 h with 5% (w/v) milk powder in 1X TBST buffer (10 mM Tris pH 7.5; 150 mM NaCl; 0.1% (v/v) Tween 20). The membrane was subsequently incubated overnight at 4 °C with a primary antibody diluted in TBST buffer. The primary antibodies used for western blot detection are described in Table 2. Next, the membrane was incubated for 1 h at RT with a fluorescence-tagged secondary antibody. The secondary antibodies IRDye^®^ 680LT goat anti-mouse (926-68020), IRDye^®^ 800CW goat anti-mouse (926-32210) and IRDye^®^ 800CW goat anti-rabbit (926-32211) (all LI-COR Biosciences, Lincoln, NE, USA) were diluted 1:5000 in TBST buffer. Immunodetection was performed using an Odyssey FC instrument and Image Studio 4.0 software (LI-COR Biosciences).

**Table 2:** Antibodies used for western blot, filter retardation assay and DD-AGE

Target protein antibody	Species	Dilution	Product number	Manufacturer
Ataxin-3 clone 1H9	mouse	1:2500	MAB5360	Merck KGaA, Darmstadt, DE
Ataxin-2	rabbit	1:5000	21776-1-AP	Proteintech group Inc. Rosemont, Illinois, USA
GAPDH	mouse	1:5000	sc-47,724	Santa Cruz Biotechnology, Dallas, USA
Beta-Actin	mouse	1:5000	A5441	Sigma-Aldrich Chemie GmbH, München, DE

### Filter retardation assay

Prior to the filter retardation assay analysis, the samples (*n* = 3) were mixed with 1X DPBS, 2% (w/v) SDS and 50 mM DTT to achieve a final protein concentration of 1 µg and then heated at 95 °C for 5 min.

The filter trap was performed according to standard protocols by using the Minifold II Slot-Blot System (Schleicher & Schuell, Düren, DE) [38]. First, a nitrocellulose membrane with a 0.45 μm pore size (Amersham Protran, Cytiva) was equilibrated twice with equilibration buffer (1X DPBS; 0.1% (w/v) SDS). Under vacuum, the samples were loaded onto the membrane, which was then washed twice with 1x DPBS. Blocking and immunodetection were performed according to the same protocol described for western blotting. The antibodies used for detection of ataxin-3 and ataxin-2 were the same as those listed in Table 2. The secondary antibodies IRDye^®^ 680LT goat anti-mouse (926-68020) and IRDye^®^ 800CW goat anti-rabbit (926-32211) were used.

### Denaturing detergent agarose gel electrophoresis (DD-AGE)

To perform DD-AGE, samples (*n* = 3) were prepared to a final total protein concentration of 25 µg in 25% (v/v) 4X DD-AGE buffer containing 2X TAE buffer (40 mM Tris, 20 mM acetic acid, 1 mM EDTA 0.1% SDS), 50% (v/v) glycerol, 8% (w/v) SDS and 0.01 g of Orange G.

DD-AGE analysis was performed according to the previously described SDD-AGE protocol [39]. Alterations to the protocol were as follows. A PerfectBlue™ Gel System Mini L (Peqlab, Erlangen, DE) agarose gel with 1% (w/v) agarose in 50 mL of 1X TAE buffer (40 mM Tris, 20 mM acetic acid, 1 mM EDTA) and 0.1% (w/v) SDS was added. The gel electrophoresis was performed in 1X TAE running buffer containing 0.1% (w/v) SDS at 40 V for approximately 2 h until the running front migrated 4 cm. The proteins were transferred onto nitrocellulose membranes following the same procedure described for western blotting, with the only modification being the addition of 10% methanol to the Bicine/Bis-Tris transfer buffer. Blocking and immunodetection were carried out as described for the western blot analysis. The antibodies used and their respective concentrations were identical to those used for filter traps and western blotting (Table 2).

### Immunohistochemistry

Seven µm-thick sagittal brain sections of 18-month-old homozygous SCA3 knock-in mice (*n* = 3) [37] were deparaffinized in xylene and an alcohol series via a Leica autostainer XL (Leica, Wetzlar, Germany). After microwave treatment (10 mM sodium citrate and 10 mM citric acid for 15 min), endogenous peroxidase activity was blocked with 1.6% peroxidase (Sigma-Aldrich, Munich, Germany). Blocking was performed in 5% normal serum (Vector Laboratories, Burlingame, USA) supplemented with 0.3% Triton X-100 (Carl Roth, Karlsruhe, Germany) for 45 min at RT. Primary antibodies (anti-Ataxin-3 clone 1H9, 1:400, Merck Millipore, Darmstadt Germany or anti-Ataxin-2, 1:500, BD Biosciences, Heidelberg, Germany) diluted in PBS and normal serum were incubated overnight at 4 °C. Next, the sections were incubated with a biotinylated secondary antibody (goat anti-mouse, 1:200, Vector Laboratories) at RT for 1 h. The slices were incubated with the avidin-biotin complex (ABC; Vector Laboratories) for two hours at RT. After washing with PBS, the substrate 3,3´-diaminobenzidine (DAB, Sigma-Aldrich) was added to the sections, and when the desired degree of staining was reached, the reaction was stopped in water. After dehydrating the slices, the sections were mounted with CV Ultra mounting media (Leica). Imaging was performed with an Axioplan imaging microscope (Zeiss).

### Statistical analysis

The western blot, filter trap and DD-AGE results were quantified using LI-COR Image Studio program (Image Studio Lite Ver 5.2; Image Studio Ver 2.1; LI-COR^®^ Odyssey^®^ Fc Imaging System).

Statistical analysis was performed with GraphPad Prism 10 (GraphPad Software Inc., San Diego, USA). The results concerning *ATXN2* CAG repeat length analysis, normality and lognormality were determined with the Shapiro-Wilk test; therefore, the Kruskal-Wallis test was used. Except for the normally distributed AAO, ordinary one-way ANOVA was performed. Furthermore, Spearman correlation and simple linear regression were performed. For the analysis of the 9bp region, the Shapiro-Wilk test was performed, and depending on the outcome, a Mann-Whitney U-test was performed, except for the normally distributed AAO; here, an unpaired t-test, Spearman correlation and simple linear regression were also performed. Two-way ANOVA was performed for the western blot, filter trap and DD-AGE results. The RNA sequencing blood and cerebellum data were analysed via two-way ANOVA and an unpaired t-test. The data are presented within the corresponding figure legend. A p-value of < 0.05 was considered to indicate statistical significance. For simplification, significant p-values are represented by asterisks (*) if not specifically indicated: * = p-value < 0.05; ** = p-value < 0.001 and *** = p-value < 0.0001.

## Results

In total, *n* = 390 probands were included in this study (demographic data presented in Table 3). An additional 12 organ donors contributed cerebellum samples for RNA sequencing and bioinformatic analysis of differential gene and isoform expression (demographic data published in [37]).

As expected, preataxic SCA3 MCs were younger than ataxic SCA3 MCs. The number of CAG repeats in *ATXN3* did not differ between preataxic and ataxic MCs (*p* = 0.3654, Table 3). The number of CAG repeats in the *ATXN2* gene did not significantly differ (*p* = 0.1481) between all genotypes. The SARA and INAS were significantly different among the three groups depending on the disease stage (Table 3).

**Table 3:** Demographic data of SCA3 study cohort used for genetic analyses and RNA sequencing in blood. Cohort was divided into healthy controls, preataxic SCA3 MC (SARA ≤ 2.5) and ataxic SCA3 MC (SARA ≥ 3), SD = standard deviation, IQR = interquartile range, sig. = significance

*N*	CNTR	SCA3 preataxic	SCA3 ataxic	sig.	*p*-value
	114	55	221		*n*.a.
N male / female (%)	48/60 (44.44/55.55)	21/34 (38.18 /61.81)	117/104 (52.94/47.59)		0.0934^1^
Age; mean [SD]	45.47 [13.52]	33.95 [8.57]	51.76 [11.30]	***	< 0.0001^2^
ATXN3 CAG repeats longer allele; median [IQR]	n.a.	69.00 [66.00, 70.00]	69.00 [66.00, 71.00]		0.3654^3^
ATXN2 CAG repeats longer allele; median [minimum, maximum]	22.00 [21, 29]	22.00 [21, 27]	22.00 [16, 30]		0.1481^4^
N 9bp wt/dup (%)	96/2 (97.96/2.04)	45/2 (95.74/4.26)	183/8 (95.81/4.17)		0.6435^1^
Age at onset; mean [SD]	n.a.	n.a.	39.91 [10.27]		n.a.
SARA sum score; median [IQR]	0.00 [0.00, 0.50]	1.00 [0.00, 2.00]	12.00 [8.00, 19.50]	***	< 0.0001^4^
INAS count; median [IQR]	0.00 [0.00, 1.00]	1.00 [0.00, 2.00]	5.00 [3.00, 7.00]	***	< 0.0001^4^
Disease duration, in years; median [IQR]	n.a.	n.a.	11.00 [6.00, 16.25]		n.a.

^1^ Fisher Test; ^2^ ANOVA; ^3^ Mann-Whitney U test; ^4^ Kruskal-Wallis Test, n.a.= not applicable; *** *p* < 0.001

### The occurrence of intermediate *ATXN2* CAG repeats leads to a significant increase in non-ataxic symptoms, including polyneuropathy symptoms

**Fig. 1:**
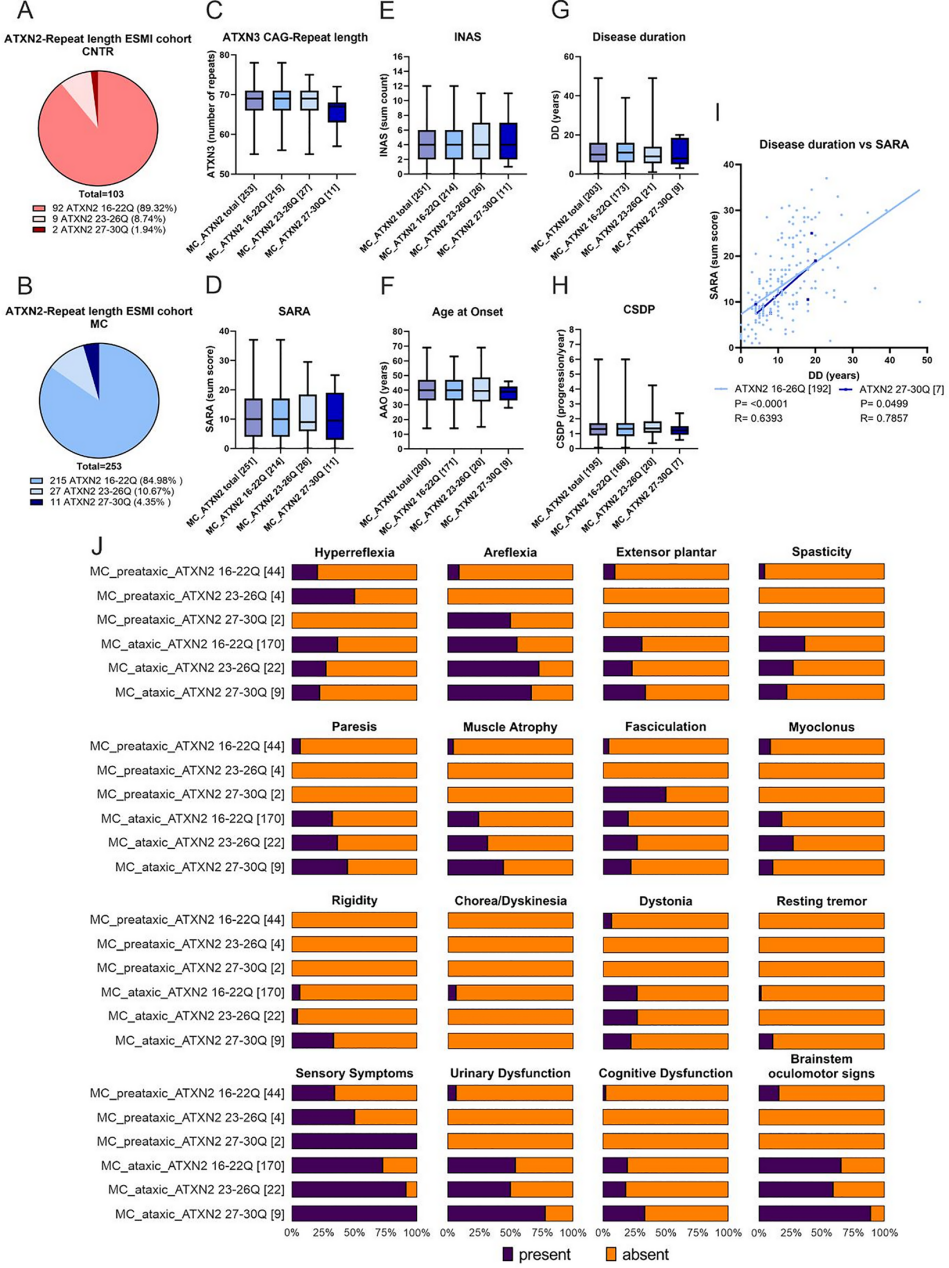
ESMI probands with an intermediate ATXN2 repeat length (27-30Q) had a non-significant lower ATXN3 CAG-repeat count and significant increase in non-ataxic symptoms, including polyneuropathy symptoms compared to SCA3 MC without an intermediate ATXN2 repeat. (**A**) Proportion of ATXN2 wildtype repeat length (16-22Q), intermediate short (23-26Q) and intermediate repeat length (27-30Q) in healthy controls (CNTR) and (**B**) in SCA3 mutation carriers (MC). **C**-**H**) Box-Whisker-Plot categorized into groups of different ATXN2 repeat lengths (all MC, 16-22Q, 23-26Q, 27-30Q). Whiskers display the minimum and maximum value, whereas the box ranges from 25th to the 75th percentiles and the line represents the median. The Kruskal-Wallis test was used for statistical analysis of **C**-**E**) and **G**-**H**). Statistical analysis of the data presented in **F**), a one-way ANOVA was applied. **I**) Correlation of SARA and DD comparing normal and intermediate ATXN2 repeat length in SCA3 MC with simple linear regression curve for non-parametric data (Spearman r). **J**) Bar chart showing frequency of individual non-ataxic symptoms (INAS subitems, present (blue) or absent (orange)). SCA3 MC were grouped by ATXN2 repeat length (16-22Q, 23-26Q and 27-30Q) and preataxic (SARA < 3) and ataxic (SARA ≥ 3) staging. MC = mutation carrier, CNTR = healthy controls, AAO = age at onset, DD = disease duration, CSDP = cross-sectional disease progression, Q = glutamine, N-numbers are indicated in brackets

A total of 356 (103 CNTR/253 SCA3 MC) blood samples were analyzed to evaluate the impact of the intermediate CAG repeat length of *ATXN2* on the clinical manifestations of SCA3 MC. In the ESMI cohort, a range between 16 and 30 *ATXN2* CAG repeats in SCA3 MC and 19 to 29 in CNTR were observed. The most frequent allele found was ATXN2 22Q (whole cohort 69.7%, MC 70.8%, CNTR 68.0%), followed by ATXN2 21Q (whole cohort 23.9%, MC 19.4%, CNTR 34.9%). Overall, 11 out of 253 (4.35%) SCA3 MCs were identified with an intermediate CAG repeat length of ATXN2 27-30Q. In the control group, only 2 out of 103 (1.94%) probands were identified with an intermediate CAG repeat (Fig. 1A, B). Among the 13 probands, one out of the 72 was English (UK), nine out of the 130 were German, and three out of the 119 were Portuguese (additional Fig. 1A-C). Among all 13 subjects with an intermediate repeat length, one sibling pair from Germany is reported (one CNTR and one SCA3 MC). No additional familial relationships are described. The correlation of clinical parameters with *ATXN2* repeat length revealed a non-significantly lower median DD and CSDP in the ATXN2 27-30Q group than in the SCA3 MC with ATXN2 16-22Q and ATXN2 23-26Q groups (Fig. 1G-H). The total SARA score (*p* = 0.9779) and total INAS count (*p* = 0.7360) did not differ between the groups (Fig. 1D-E). SCA3 MC with an intermediate repeat (ATXN2 27-30Q) revealed a one-year earlier AAO (median 39.0 years) compared to subjects with shorter *ATXN2* CAG repeat length (ATXN*2* 16-22Q: AAO median 40.0 years; ATXN2 23-26Q: AAO mean 39.5 years, Fig. 1F). Additionally, a non-significantly shorter *ATXN3* CAG repeat length was detected for SCA3 MC *ATXN2* 27-30Q (mean = 65.64) than for *ATXN2* 16-22Q (mean = 68.53) and *ATXN2* 23-26Q (mean = 67.78) (Kruskal-Wallis test *p* = 0.0799, Fig. 1C). Therefore, the slightly earlier AAO of ATXN2 27-30Q contrasts with the fact that this group had a shorter *ATXN3* repeat length than the other groups, which would normally suggest a later AAO. Simple linear regression analysis (Spearman r) demonstrated that SCA3 MCs harbouring an *ATXN2* 16-26Q had higher SARA scores and a longer DD (*r* = 0.6393 and p-value of < 0.0001) compared to SCA3 MC with an intermediate *ATXN2* repeat (*r* = 0.7857, *p* = 0.0499 (Fig. 1I). Next, INAS subcategories indicating non-ataxic symptoms were correlated with the repeat length within *ATXN2* (Fig. 1J). INAS items related to polyneuropathy were more common in SCA3 MCs with an intermediate *ATXN2* CAG repeat (Fig. 1J; Table 4). Compared with SCA3 MCs with normal *ATXN2* repeat length 16-22Q, all preataxic and ataxic MCs with intermediate repeat lengths presented sensory symptoms (Fig. 1I; Table 4). A closer look at the ataxic probands revealed a significant increase in the occurrence of sensory symptoms (*p* = 0.0319) compared with all groups with different *ATXN2* CAG repeat lengths (Table 5). Furthermore, SCA3 MCs with a short-intermediate length (ATXN2 23-26Q) presented sensory problems more often than SCA3 MCs with *ATXN2* 16-22Q, but fewer than MCs with long intermediate repeat (≥ 27 CAG) (Fig. 1J; Table 5). With respect to parkinsonian symptoms, there was a significant increase in rigidity (*p* = 0.0402) and a tendency toward a non-significant increase in resting tremor (*p* = 0.2230) when the ataxic SCA3 MC with *ATXN2* 27-30Q were compared with that without intermediate *ATXN2* repeats (Fig. 1J; Table 5). Spasticity and hyperreflexia were found less frequently in the ataxic groups with intermediate *ATXN2* repeat lengths (Fig. 1J; Table 5), although the differences did not reach statistical significance (*p* = 0.5572).

**Table 4:** INAS sum count analysis of individual INAS item in ATXN2 CAG repeat cohort, divided by preataxic (SARA < 3) and ataxic (SARA *≥* 3), as well as their ATXN2 repeat length (16-22Q, 23-26Q and 27-30Q). Here, absolute values (n present/ n absent) and percentages of presence/absence in brackets of symptoms for each category are presented. Statistical analyses using fisher test

INAS item	ATXN2 CAG repeat length in SCA3 MC [*N* present / absent (%/%)]						sig.	*p*-value
N present/ N absent (% present/% absent)	***Preataxic*** ***16-22Q***	***Preataxic*** ***23-26Q***	***Preataxic*** ***27-30Q***	***Ataxic*** ***16-22Q***	***Ataxic*** ***23-26Q***	***Ataxic*** ***27-30Q***		
N	44	4	2	170	22	9		
Hyperreflexia	9/35 (20.5/79.5)	2/2 (50.0/50.0)	0/2 (0.0/100.0)	62/108 (36.5/63.5)	6/16 (27.3/72.7)	2/7 (22.2/77.8)		0.2748
Areflexia	4/40 (9.1/90.9)	0/4 (0.0/100.0)	1/1 (50.0/50.0)	94/76 (55.3/44.7)	16/6 (72.7/27.3)	6/3 (66.7/33.3)	***	**< 0.0001**
Extensor plantar	4/40 (9.1/90.9)	0/4 (0.0/100.0)	0/2 (0.0/100.0)	52/118 (30.6/69.4)	5/17 (22.7/77.3)	3/6 (33.3/66.7)	***	**< 0.0001**
Spasticity	2/42 (4.5/95.5)	0/4 (0.0/100.0)	0/2 (0.0/100.0)	62/108 (36.5/63.5)	6/16 (27.3/72.7)	2/7 (22.2/77.8)	**	**0.0001**
Paresis	3/41 (6.8/93.2)	0/4 (0.0/100.0)	0/2 (0.0/100.0)	55/115 (32.4/63.6)	8/14 (36.4/63.6)	4/5 (44.4/55.6)	**	**0.0021**
Muscle Atrophy	2/42 (4.5/95.5)	0/4 (0.0/100.0)	0/2 (0.0/100.0)	42/128 (24.7/75.3)	7/15 (31.8/68.2)	4/5 (44.4/55.6)	**	**0.0054**
Fasciculation	2/42 (4.5/95.5)	0/4 (0.0/100.0)	1/1 (50.0/50.0)	34/136 (20.0/80.0)	6/16 (27.3/72.7)	2/7 (22.2/77.8)	*	**0.0398**
Myoclonus	4/40 (9.1/90.9)	0/4 (0.0/100.0)	0/2 (0.0/100.0)	31/139 (18.2/81.8)	6/16 (27.3/72.7)	1/8 (11.1/88.9)		0.4431
Rigidity	0/44 (0.0/100.0)	0/4 (0.0/100.0)	0/2 (0.0/100.0)	11/159 (6.5/93.5)	1/21 (4.5/95.5)	3/6 (33.3/66.7)	*	**0.0321**
Chorea and Dyskinesia	0/44 (0.0/100.0)	0/4 (0.0/100.0)	0/2 (0.0/100.0)	11/159 (6.5/93.5)	0/22 (0.0/100.0)	0/9 (0.0/100.0)		0.4390
Dystonia	3/41 (6.8/93.2)	0/4 (0.0/100.0)	0/2 (0.0/100.0)	46/124 (27.1/72.9)	6/16 (27.3/72.7)	2/7 (22.2/77.8)	*	**0.0444**
Resting Tremor	0/44 (0.0/100.0)	0/4 (0.0/100.0)	0/2 (0.0/100.0)	3/167 (1.8/98.2)	0/22 (0.0/100.0)	1/8 (11.1/88.9)		0.2929
Sensory symptoms	15/29 (34.1/65.9)	2/2 (50.0/50.0)	2/0 (100.0/0.0)	123/47 (72.4/27.6)	20/2 (90.9/9.1)	9/0 (100.0/0.0)	***	**< 0.0001**
Urinary dysfunction	3/41 (6.8/93.2)	0/4 (0.0/100.0)	0/2 (0.0/100.0)	92/78 (54.1/45.9)	11/11 (50.0/50.0)	7/2 (77.8/22.2)	***	**< 0.0001**
Cognitive dysfunction	1/43 (2.3/97.7)	0/4 (0.0/100.0)	0/2 (0.0/100.0)	33/137 (19.4/80.6)	4/18 (18.2/81.8)	3/6 (33.3/66.7)	*	**0.0245**
Brainstem oculomotor signs	7/37 (15.9/84.1)	0/4 (0.0/100.0)	0/2 (0.0/100.0)	111/59 (65.3/34.7)	13/9 (59.1/40.9)	8/1 (88.9/11.1)	***	**< 0.0001**

Fisher Test * *p* < 0.05, ** *p* < 0.001, *** *p* < 0.0001

**Table 5:** INAS sum count, statistical evaluation of individual INAS item in ATXN2 CAG repeat cohort, for only ataxic SCA3 MC, divided by their ATXN2 repeat length (16-22Q, 23-26Q and 27-30Q). Data in relation to Table 4. Statistical analyses using the fisher test comparing SCA3 ataxic MC with the different ATXN2 repeat length in respect to presence/ absence of INAS subcategories

ATXN2 CAG repeat length in ataxic SCA3 MC	Item	sig.	*p*-value
*16-22Q*,* 23-26Q and 27-30Q*	Hyperreflexia		0.5572
	Areflexia		0.2767
	Extensor plantar		0.7939
	Spasticity		0.5572
	Paresis		0.6530
	Muscle Atrophy		0.2827
	Fasciculation		0.6951
	Myoclonus		0.6152
	**Rigidity**	*	**0.0402**
	Chorea and Dyskinesia		0.7725
	Dystonia		> 0.9999
	Resting Tremor		0.2230
	**Sensory symptoms**	*	**0.0319**
	Urinary dysfunction		0.3659
	Cognitive dysfunction		0.5446
	Brainstem oculomotor signs		0.2925

Fisher Test * *p* < 0.05

### Occurrence of 9bp duplication in *ATXN2* leads to non-significant later AAO with faster disease progression

**Fig. 2:**
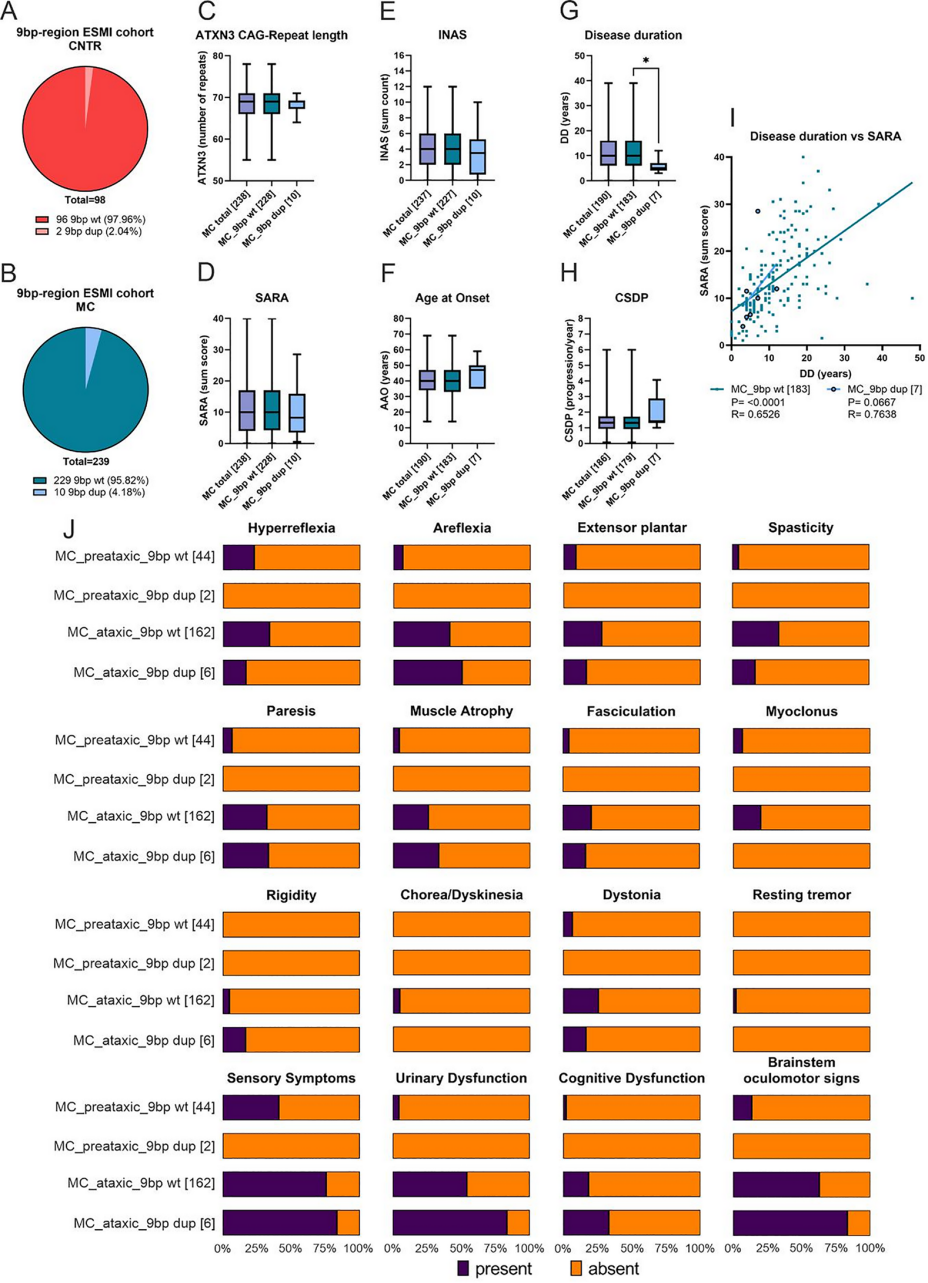
ESMI probands with a 9bp dup in ATXN2 showed a significantly reduced DD as well as a greater AAO and CSDP compared to SCA3 MC without 9bp dup (9bp wt). **A**) Proportion of ATXN2 9bp wt and 9bp dup in CNTR and **B**) in SCA3 MC. **C**-**H**) Box-Whisker-Plot demonstrate correlation of clinical data, categorized into groups, all SCA3 MC, SCA3 MC with 9bp wt and SCA3 MC with 9bp dup. Whiskers display the minimum and maximum value, whereas the box ranges from 25th to the 75th percentiles and the line represents the median. **C**-**E**) and **G**-**I**) The Kruskal-Wallis test was used for statistical analysis between all SCA3 MC, SCA3 MC with 9bp wt and SCA3 MC with 9bp dup. Mann-Whitney test to compare SCA3 MC with 9bp wt and 9bp dup. **F**) An ordinary one-way ANOVA was applied for the statistical evaluation. **I**) Correlation of SARA and DD comparing SCA3 MC without and with 9bp dup using a simple linear regression curve for non-parametric data (Spearman r). **J**) Bar chart showing the frequency of individual non-ataxic symptoms (INAS subitems, present (blue) or absent (orange)). SCA3 MC were grouped by 9bp region properties (9bp wt, 9bp dup) and preataxic (SARA < 3) and ataxic (SARA ≥ 3) staging. SCA3 MC = mutation carrier, CNTR = healthy controls, wt = wildtype, dup = duplication, AAO = age at onset, DD = disease duration, CSDP = cross-sectional disease progression, Q = glutamine, N-numbers are indicated in brackets, * *p* < 0.05

To detect the frequency of the 9bp duplication in *ATXN2* described by [32] in our large European cohort, a total of 337 blood samples (98 CNTR and 239 SCA3 MC) were analyzed. In healthy controls, only two (2.04%) individuals with the 9bp dup were identified, whereas in SCA3 MC, 10 (4.18%) individuals were found with this genetic variant (Fig. 2A, B). The geographical distribution of these probands was as follows: nine (75.0%) from Germany, two (16.67%) from Portugal, and one (8.33%) from The Netherlands (additional Fig. 2A-B). No familial relationship was reported among SCA3 MCs with a 9bp dup in our cohort. Importantly, no 9bp dup was detected in individuals harboring an intermediate *ATXN2* CAG repeat (as described in Fig. 1). Statistical analyses revealed no differences in *ATXN3* CAG repeat length (Mann Whitney U-test *p* = 0.6052), SARA sum score (*p* = 0.6830), and INAS sum count (*p* = 0.4090) in SCA3 patients who harbored the 9bp dup compared with SCA3 patients without the 9bp dup (9bp wt, Fig. 2C-E). However, there was a significantly lower DD in the SCA3 MC with 9bp dup than in the SCA3 MC 9bp wt (*p* = 0.0260) (Fig. 2G), although the mean age at data collection was rather similar in both groups (9bp wt: 48.3 years and 9bp dup: 48.6 years; data not shown). In contrast to an earlier study [32], our cohort showed a non-significant greater median AAO (*p* = 0.2406) and a non-significantly faster CSDP (*p* = 0.2214) (Fig. 2F-H). The faster disease progression in SCA3 MC with 9bp dup compared to SCA3 MC with 9bp wt was also visible in the corresponding simple linear correlation analyses (Spearman rho; MC_9bp wt p = < 0.0001, *r* = 0.6526; MC_9bp dup *p* = 0.0667, *r* = 0.7638) (Fig. 2I). The occurrence of INAS subitems was not significantly different between ataxic SCA3 MCs with 9bp dup and MCs with 9bp wt SCA3 MCs (Fig. 2J; Table 7). Hyperreflexia and spasticity were less common in the 9bp dup probands than in the 9bp wt probands (Fig. 2J; Table 6). However, areflexia, rigidity and brainstem oculomotor signs were found more often in SCA3 MCs with 9bp dup in contrast to 9bp wt (Fig. 2J; Table 6). Concerning the symptoms related to polyneuropathy (sensory symptoms, paresis and muscle atrophy) and parkinsonism (rigidity, resting tremor), no significant difference in the ataxic cohort between 9bp dup and 9bp wt groups was found (Table 7).

**Table 6:** INAS sum count analysis of individual INAS item in ATXN2 9bp cohort, divided by preataxic (SARA < 3) and ataxic (SARA *≥* 3), as well as their 9bp status (wt or dup). Table demonstrates absolute values and percentages of presence/absence of symptoms for each category. Here, absolute values (n present/ n absent) and percentages of presence/absence in brackets of symptoms for each category are presented. Statistical analyses using fisher test

INAS item	ATXN2 9bp wt / dup in SCA3 MC [*N* present / absent (%)]					sig.	*p*-value
*N* present/ *N* absent (% present/% absent)	Preataxic 9bp wt	Preataxic 9bp dup	Ataxic 9bp wt		Ataxic 9bp dup		
N	44	2	162		6		
Hyperreflexia	10/34 (22.7/77.3)	0/2 (0.0/100.0)	62/121 (33.9/66.1)	1/5 (16.7/83.3)			0.3988
Areflexia	3/41 (6.8/93.2)	0/2 (0.0/100.0)	75/108 (41.0/59.0)	3/3 (50.0/50.0)		***	**< 0.0001**
Extensor plantar reflex	4/40 (9.1/90.9)	0/2 (0.0/100.0)	51/132 (27.9/72.1)	1/5 (16.7/83.3)		*	**0.0335**
Spasticity	2/42 (4.5/95.5)	0/2 (0.0/100.0)	62/121 (33.9/66.1)	1/5 (16.7/83.3)		***	**< 0.0001**
Paresis	3/41 (6.8/93.2)	0/2 (0.0/100.0)	59/124 (32.2/67.8)	2/4 (33.3/66.7)		**	**0.0015**
Muscle Atrophy	2/42 (4.5/95.5)	0/2 (0.0/100.0)	47/136 (25.7/74.3)	2/4 (33.3/66.7)		**	**0.0041**
Fasciculation	2/42 (4.5/95.5)	0/2 (0.0/100.0)	38/145 (20.8/79.2)	1/5 (16.7/83.3)		*	**0.0474**
Myoclonus	3/41 (6,8/93.2)	0/2 (0.0/100.0)	37/146 (20.2/79.8)	0/6 (0.0/100.0)			0.1093
Rigidity	0/44 (0.0/100.0)	0/2 (0.0/100.0)	9/174 (4.9/95.1)	1/5 (16.7/83.3)			0.1635
Chorea and Dyskinesia	0/44 (0.0/100.0)	0/2 (0.0/100.0)	9/174 (4.9/95.1)	0/6 (0.0/100.0)			0.4262
Dystonia	3/41 (6.8/93.2)	0/2 (0.0/100.0)	47/136 (25.7/74.3)	1/5 (16.7/83.3)		*	**0.0239**
Resting Tremor	0/44 (0.0/100.0)	0/2 (0.0/100.0)	4/179 (2.2/97.8)	0/6 (0.0/100.0)			> 0.9999
Sensory symptoms	18/26 (40.9/59.1)	0/2 (0.0/100.0)	138/45 (75.4/24.6)	5/1 (83.3/16.7)		***	**< 0.0001**
Urinary dysfunction	2/42 (4.5/95.5)	0/2 (0.0/100.0)	99/84 (54.1/45.9)	5/1 (83.3/16.7)		***	**< 0.0001**
Cognitive dysfunction	1/43 (2.3/97.7)	0/2 (0.0/100.0)	34/149 (18.6/81.4)	2/4 (33.3/66.7)		*	**0.0101**
Brainstem oculomotor signs	6/38 (13.6/86.4)	0/2 (0.0/100.0)	115/68 (62.8/37.2)	5/1 (83.3/16.7)		***	**< 0.0001**

Fisher Test * *p* < 0.05, ** *p* < 0.001, *** *p* < 0.0001

**Table 7:** INAS sum count, statistical evaluation of individual INAS item in ATXN2 9bp cohort, for only ataxic SCA3 MC, divided by their 9bp duplication (wt, dup). Data in relation to Table 6. Statistical analyses using the fisher test comparing SCA3 ataxic MC with (dup) or without (wt) 9bp dup in respect to presence/ absence of INAS subcategories

ATXN2 9bp region in ataxic SCA3 MC	Item	sig.	*p*-value
*9bp wt and 9bp dup*	Hyperreflexia		0.6654
	Areflexia		0.6922
	Extensor plantar		> 0.9999
	Spasticity		0.6654
	Paresis		> 0.9999
	Muscle Atrophy		0.6503
	Fasciculation		> 0.9999
	Myoclonus		0.5994
	Rigidity		0.2816
	Chorea and Dyskinesia		> 0.9999
	Dystonia		> 0.9999
	Resting Tremor		> 0.9999
	Sensory symptoms		> 0.9999
	Urinary dysfunction		0.2256
	Cognitive dysfunction		0.3209
	Brainstem oculomotor signs		0.2925

Fisher Test

### Bioinformatic analysis of *ATXN2* isoform expression in the cerebellum and blood revealed altered splicing profiles in different tissues, with significantly higher expression of *ATXN2* isoforms in SCA3 MC than in CNTR and overall low expression of protein-coding isoforms carrying the CAG repeat

**Fig. 3:**
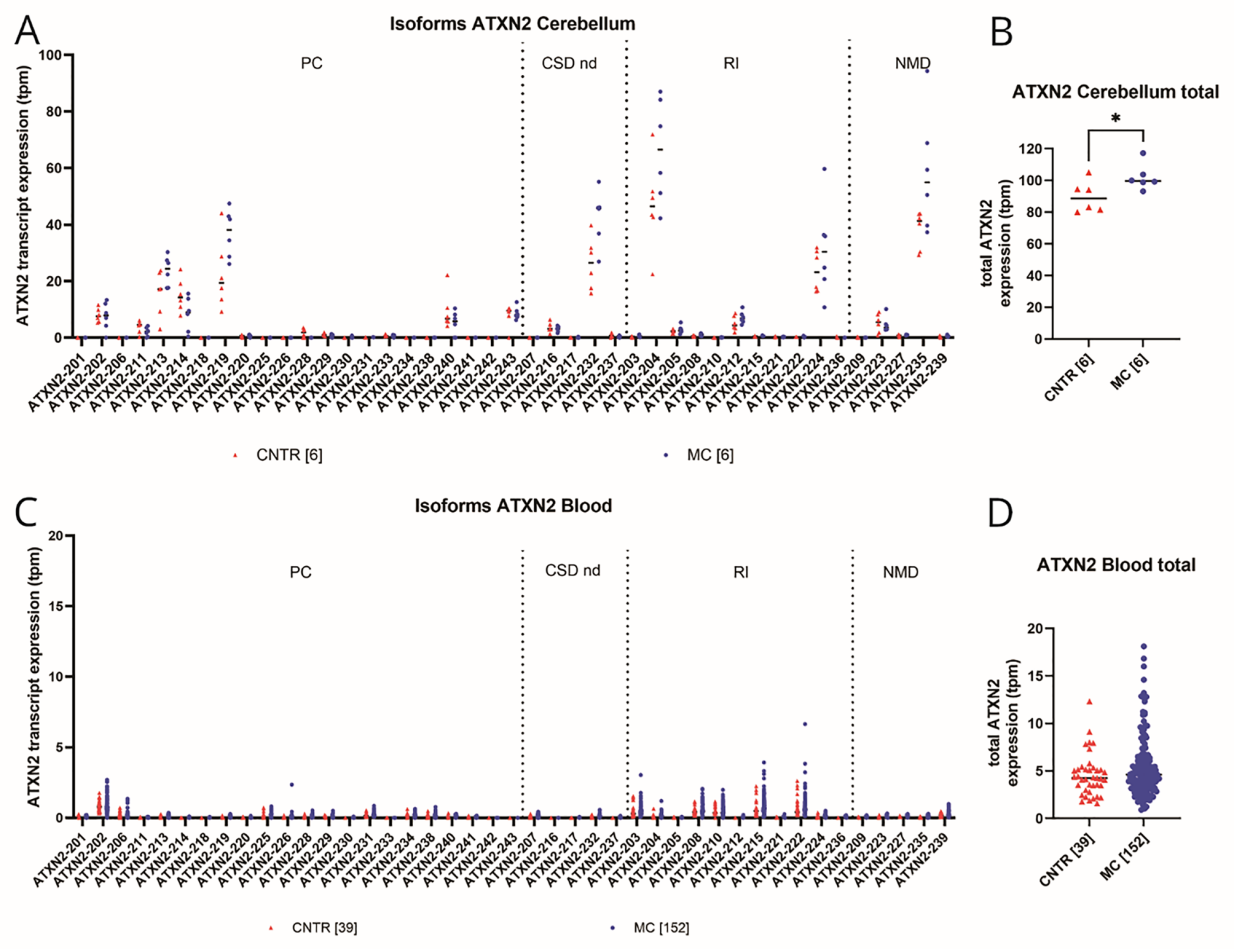
Bioinformatic analysis of RNA sequencing data from post-mortem cerebellar and blood samples demonstrates significant different total ATXN2 expression in SCA3 mutation carriers (SCA3 MC) compared to healthy controls (CNTR) and a different isoform profile between brain and blood. **A**, **B**) ATXN2 total and isoform expression in post-mortem cerebellar samples, *n* = 6 per genotype. **A**) Scatter dot plot displays ATXN2 isoform expression in transcript per million (tpm). ATXN2 isoforms categorized into Ensembl biotypes and divided into controls (red) and SCA3 MC (blue). **B**) Scatter plot of total ATXN2 expression in tpm. Statistical analysis using an unpaired t-test revealed a significant greater ATXN2 RNA expression in SCA3 MC compared to CNTR. **C**, **D**) ATXN2 total and isoform expression in blood samples from *n* = 39 CNTR and *n* = 152 SCA3 MC. **C**) Graph shows all known ATXN2 isoforms in tpm and categorized into Ensembl biotypes. Controls are displayed in red and SCA3 MC in blue. **D**) Scatter plot of total ATXN2 expression in tpm, compared in CNTR and SCA3 MC. Analysis with unpaired t-test. CNTR = Control group, SCA3 MC = SCA3 mutation carrier group, PC = protein-coding, CDS nd = protein-coding, coding sequence not defined, RI = retained intron, NMD = nonsense mediated decay, tpm = transcript per million, * *p* < 0.05

To investigate *ATXN2* isoforms in brain tissue and blood, our RNA sequence data were compared with published data displayed on Ensembl.org (human (GRCh38.p14)) (Fig. 3) [33]. *ATXN2* isoforms were determined via RNA sequencing data from 12 post-mortem cerebellar samples (6 CNTR/6 SCA3 MCs, published in [37]). The total RNA expression of cerebellar *ATXN2* was significantly greater in SCA3 MCs compared to CNTR (unpaired t-test, *p* = 0.0400) (Fig. 3B). The isoforms with the highest transcript per million (tpm) count included mostly nonprotein-coding isoforms (*ATXN2*-232, *ATXN2*-204, *ATXN2*-224, *ATXN2*-235) and only one protein-coding isoform (*ATXN2*-219) (Fig. 3A). Importantly, all these isoforms do not contain the CAG repeat and are shorter (430–1444 bp) than the reference isoform *ATXN2*-225 (4376 bp) (Ensembl.org: human (GRCh38.p14)) [33]. A closer look revealed that out of all the detected isoforms, only five contained the CAG repeat (*ATXN2*-229, *ATXN2*-231, *ATXN2*-233, *ATXN2*-240, *ATXN2*-243), which showed rather low expression in the global comparison to all *ATXN2* isoforms detected in the cerebellum (Fig. 3A). The reference isoforms *ATXN2*-225 and *ATXN2*-238 were not detected in the cerebellum (Fig. 3A). Similar to the results of the cerebellar transcriptomic analyses, 191 blood samples (39 CNTR/152 SCA3 MC) from the ESMI cohort were analyzed by RNA sequencing. The total *ATXN2* RNA levels were lower in the blood than in the brain, and the pattern of expression of the individual *ATXN2* isoforms also varied (attached Excel file) (Fig. 3A-D). The analysis of total *ATXN2* expression in blood revealed a non-significantly greater level of *ATXN2* expression in SCA3 MCs than in CNTRs (Mann-Whitney U-test, *p* = 0.2164) (Fig. 3D).

### Bioinformatic analysis of *ATXN2* isoform expression in the cerebellum and blood revealed altered splice variant profiles in different tissues, with ATXN2 splice variant type I occurring significantly lower in brain tissue of SCA3 MCs and CNTR and significantly higher in blood tissue of SCA3 MC compared to ATXN2 splice variant type II

**Fig. 4:**
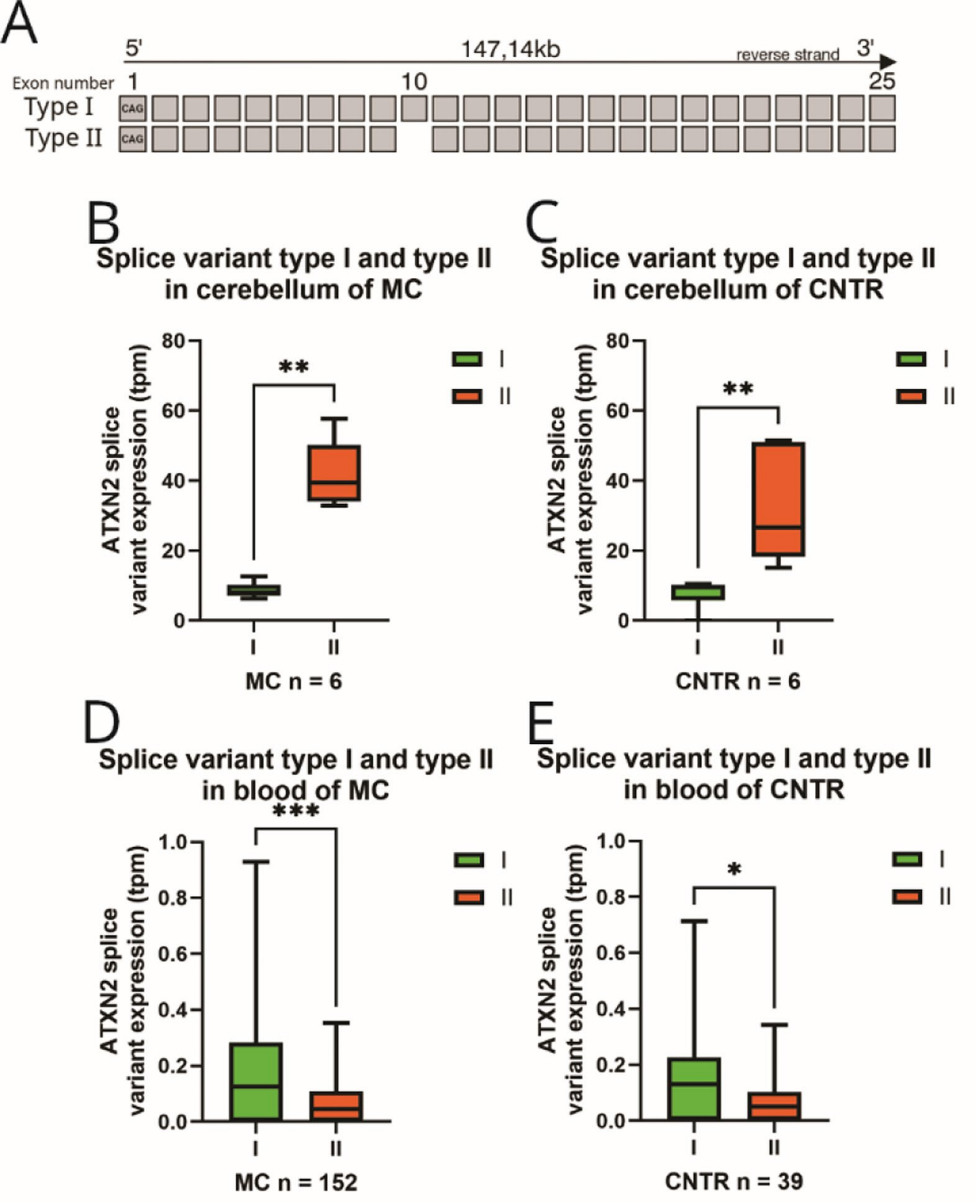
Comparison of ATXN2 splice variant type I and II in blood and cerebellar tissue analyzing expression changes in CNTR and SCA3 MC. In cerebellar tissue, splice variant type I was detected significantly lower compared to type II, whereas in blood, variant type I was expressed significantly greater compared to type II. **A**) Schematic representation of known transcripts of splice variant type I and II. **B**, **C**) Box-Whisker-Plot of total expression of splice variant I and II in cerebellar tissue for CNTR and SCA3 MC group (*n* = 6 per genotype). **D**, **E**) Box-Whisker-Plot of total expression of splice variant I and II of blood samples in CNTR and SCA3 MC group (CNTR *n* = 36, MC *n* = 152). Whiskers display the minimum and maximum value, whereas the box ranges from 25th to the 75th percentiles and the line represents the median. Statistical evaluation using Mann-Whitney test. CNTR = Control group, SCA3 MC = SCA3 mutation carrier group, tpm = transcript per million, * *p < 0.05*, ** *p<0.01*, *** *p < 0.001*

In the literature, *ATXN2* isoforms are grouped into splice variants type I-VI according to the lack of different exons compared to the full length variant I [40, 41, 42]. The splice variant I corresponds to an isoform with 25 exons containing the CAG repeat [40, 41] (Fig. 4A). This includes *ATXN2*-*218*,* ATXN2-241* and *ATXN2-243* isoforms as well as reference isoform *ATXN2*-225 and *ATXN2*-238 [33].

Splice variant type II, was described lacking exon 10 (Fig. 4A) [40, 42]. According to Ensembl.org [33], only one full-length isoform, containing a CAG repeat, which does not contain exon 10, has been described. However, this isoform, known as ATXN2-240, lacks exon 10 and exon 21 [33]. There is also a shorter protein-coding isoform lacking exon 10, *ATXN2*-219, but in total this isoform expresses only exon 7–9 + 11 + 13 and therefore does not contain the CAG region of exon 1 [33]. Our data demonstrated that splice variants of type II were found in both tissue types, whereas splice variants of type I were very low expressed (Fig. 4B-E). Comparing ATXN2 splice variants I and II, significantly higher expression levels were detected in cerebellar samples from CNTR (Mann-Whitney U-test *p* = 0.0022) and SCA3 MCs (Mann-Whitney U-test, *p* = 0.0022) of type II compared to splice variant type I (Fig. 4B, C). In blood, the opposite occurred, type I was significantly higher expressed than type II which was determined for both comparative groups, the CNTR group (Mann-Whitney U-test, *p* = 0.0408) and SCA3 MC group (Mann-Whitney U-test, p = < 0.001) (Fig. 4D, E).

Another known splice variant (type IV) lacking exon 21 has been described [41]. Among the protein-coding isoforms, type IV corresponds to seven Ensembl annotated isoforms, *ATXN2*-213, *ATXN2*-226, *ATXN2*-228, *ATXN2*-231, *ATXN2*-233, *ATXN2*-234, *ATXN2*-240, which all lack exon 21 but also additional exons. Our data presented no significant difference between CNTR and SCA3 MCs in cerebellar samples (unpaired t-test, *p* = 0.6078) or blood samples (Mann-Whitney U-test, *p* = 0.2428) (data not shown) for type IV.

The type V variant, lacking exon 12 [40], corresponds to the CAG repeat-containing and protein-coding isoform *ATXN2*-201, which was not detected in the cerebellum but was expressed in blood, with no significant difference between SCA3 MC and CNTR (data not shown).

The previously described type VI variant lacks exon 24 [40]. This variant corresponds to several short protein-coding *ATXN2* isoforms not harboring the CAG repeat of exon 1, which includes *ATXN2*-202, -211, -220, -228 and − 230 [33]. For type VI, there was no significant difference between CNTR and SCA3 MCs in cerebellar tissue (unpaired t-test, *p* = 0.1104) and blood (Mann-Whitney U-test, *p* = 0.8601) (data not shown).

### Co-expression of ataxin-2 and ataxin-3 in HEK293T cells influences ataxin-3 abundance

An earlier study demonstrated that, in SCA3 patients and SCA3 animal models, the level of soluble ataxin-2 is reduced and that re-establishment of ataxin-2 leads to a reduced level of mutant ataxin-3 as well as reduced behavioral defects and neuropathology in SCA3 mice [43]. Therefore, we analyzed the abundance of soluble ataxin-2 in our SCA3 knock-in mice [37]. Western blot analyses revealed similar levels of soluble ataxin-2 in wildtype (WT/WT), heterozygous (WT/304Q), and homozygous (304Q/304Q) SCA3 knock-in mice at the ages of 3 (additional Fig. 3), 12, and 18 months (data not shown). Importantly, age-dependent comparisons of soluble ataxin-2 in wildtype (WT/WT) and homozygous SCA3 ki mice (304Q/304Q) revealed a genotype-independent significant reduction in ataxin-2 over the whole life span (additional Fig. 4). Additionally, aggregate analyses by immunohistochemistry and DD-AGE revealed that ataxin-2 was not co-localized into ataxin-3-containing aggregates in this SCA3 mouse model (additional Fig. 5).

**Fig. 5:**
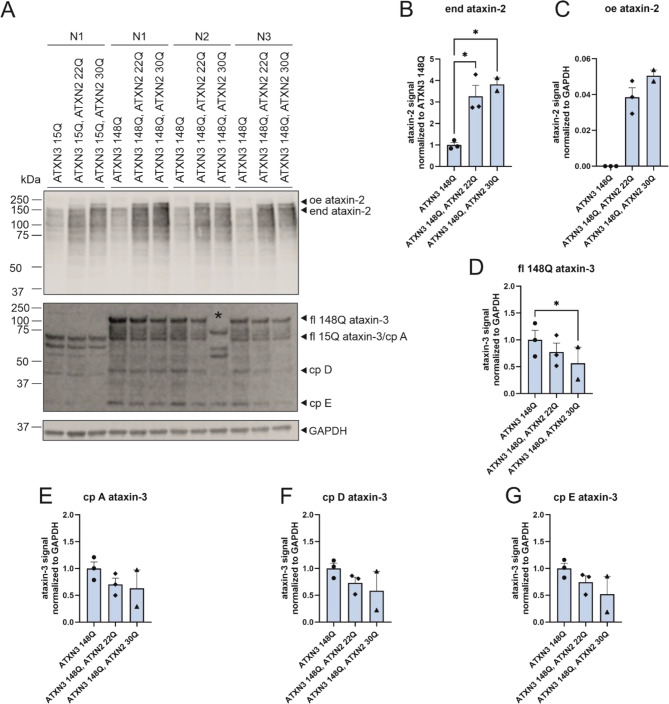
Co-expression of ataxin-2 with an intermediate CAG repeat (30Q) and an expanded ataxin-3 repeat (148Q) in HEK293T cells revealed a significant lower soluble abundance of ataxin-3 148Q. (**A**) Western blot analysis of ataxin-2 and ataxin-3 abundance in HEK293T cell lysates co-expressing ataxin-3 148Q and ataxin-2 22Q or 30Q, respectively. The sample marked with a * do not overexpress ataxin-3 148Q and therefore, was not included in any further analysis. *n* = 2–3 per construct. B-G) Bar charts, demonstrate plotted mean with SEM. Statistical analyses were performed using two-way ANOVA. (**B**) Statistical quantification demonstrates higher abundance of endogenous ataxin-2 in cells transfected with different ATXN2 constructs (ATXN2 22Q and ATXN2 30Q). (**C**) Statistical evaluation of overexpressed (oe) ataxin-2 with different repeat length. (**D**) Statistical evaluation demonstrates significant lower abundance of full-length (fl.) ataxin-3 148Q, when an intermediate ataxin-2 (30Q) repeat was co-expressed. **E**-**F**) Non-significant lower abundance of different ataxin-3 cleavage products (cp. A, D, E) influenced by different ataxin-2 repeat lengths. oe = overexpressed, end = endogenous, fl. = full-length, cp. = cleavage products, Q = glutamine, * *p* < 0.05

**Fig. 6:**
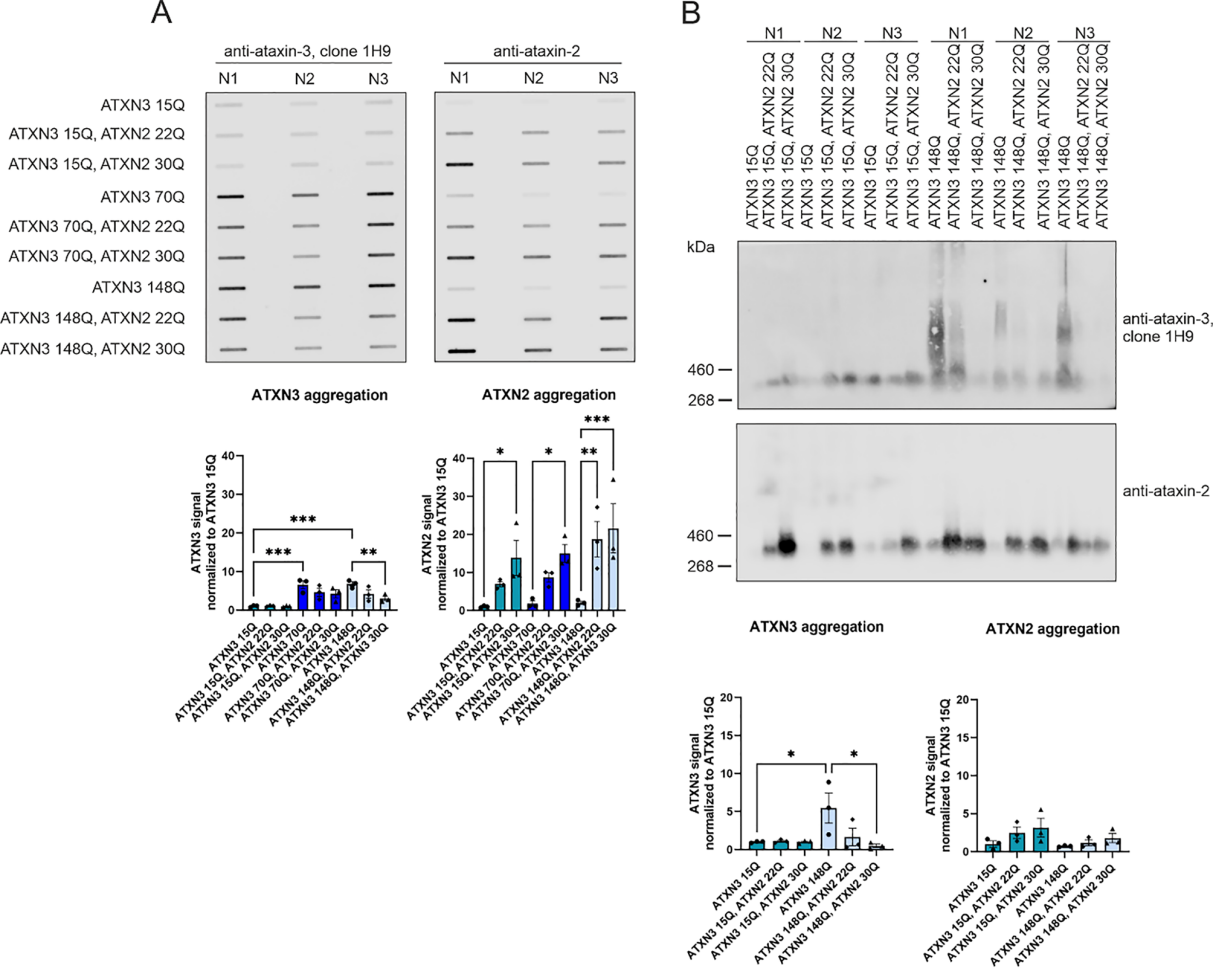
Cellular ataxin-3 protein abundance showed less ataxin-3 positive aggregates when co-expressed with ataxin-2 intermediate repeat length. (**A**) HEK293T cells were single transfected with ATXN3 15Q, 70Q or 148Q, or co-transfected with ATXN2 22Q or ATXN2 30Q, respectively. Aggregation of ataxin-3 and ataxin-2 were analysed by Filter Retardation Assay. Bar charts demonstrate plotted mean with SEM. Statistical quantification using two-way ANOVA revealed ataxin-3 aggregation influenced by ataxin-3 repeat length (15Q, 70Q, and 148Q). Additionally, ataxin-2 specific aggregation was observed in all cells which overexpressed ataxin-2 22Q and 30Q, respectively. *n* = 3 per construct. (**B**) DD-AGE stained with ataxin-3 antibody clone 1H9 revealed a high molecular weight smear in all ATXN3 148Q and transfected cells with highest aggregation load in single transfected HEK293T cells (ATXN3 148Q). Staining with an ataxin-2 specific antibody revealed only small ataxin-2 specific aggregates when ataxin-2 was co-expressed. Bar charts with plotted mean with SEM. A two-way ANOVA revealed statistically significant decreased ataxin-3 aggregation and a non-significant increased ataxin-2 aggregation if ataxin-2 with 22Q and 30Q was co-expressed. *n* = 3 per construct. Q = glutamine, * *p*<0.05, ** *p*<0.01, *** *p*<0.001

To further analyze the influence of ataxin-2 with an intermediate CAG repeat on the expression and aggregation of ataxin-3, cell culture experiments were performed using HEK293T cells transfected with ATXN3 15Q (normal repeat length) or elongated repeat length (70 and 148 CAG repeats). Additionally, cells were co-transfected with ATXN2 with a normal (22 CAG) or intermediate (30 CAG) repeat length. Western blot analyses and statistical evaluation revealed successful overexpression of ataxin-3 and ataxin-2, respectively (Fig. 5A, C, D). Interestingly, statistical quantification revealed increased endogenous ataxin-2 expression (Fig. 4B) and lower protein levels of full-length (fl.) ataxin-3 and corresponding ATXN3 cleavage products (cp.) A, D and E (Fig. 5A, D-G) when ATXN2 22Q or ATXN2 30Q was co-expressed. Importantly, the decrease in the full-length of ATXN3 and the corresponding cleavage products was the strongest between ATXN3 148Q without oe ATXN2 and ATXN3 148Q with oe ATXN2 30Q (*p* = 0.0148), indicating that an intermediate repeat led to greater downregulation of ataxin-3 and the corresponding cleavage products (Fig. 5A, D-G). Aggregate analyses via a filter retardation assay and DD-AGE revealed a decrease in ATXN3 148Q aggregate amount when the cells were also co-transfected with ATXN2 (Fig. 6A, B). A significant decrease in ataxin-3-positive aggregates in HEK293T cells transfected with ATXN3 148Q was observed when ataxin-2 was co-expressed with an intermediate repeat (filter retardation assay: *p* = 0.005, DD-AGE *p* = 0.0213). Ataxin-2 aggregation was observed in all HEK293T cells co-transfected with ATXN2 22Q or 30Q, whereas significantly more ataxin-2-positive aggregates were observed when ataxin-2 with an intermediate repeat was co-expressed (ATXN3 148Q vs. ATXN3 148Q, ATXN2 22Q *p* = 0.0045 and ATXN3 148Q vs. ATXN3 148Q, ATXN2 22Q *p* = 0.0010, Fig. 6A). DD-AGE analyses, which separated aggregates by size in an agarose gel, revealed high-molecular-weight ataxin-3 aggregates (mostly larger than 460 kDa), whereas ataxin-2-specific aggregates were smaller in size (approximately between 260 and 460 kDa) (Fig. 6B). Importantly, ataxin-3 and ataxin-2 aggregation patterns differ in size and amount, revealing that ataxin-2 and ataxin-3 potentially do not co-localizing within the same aggregates, as also indicated by immunohistochemistry in SCA3 knock-in mice (Additional Fig. 5).

**Fig. 7:**
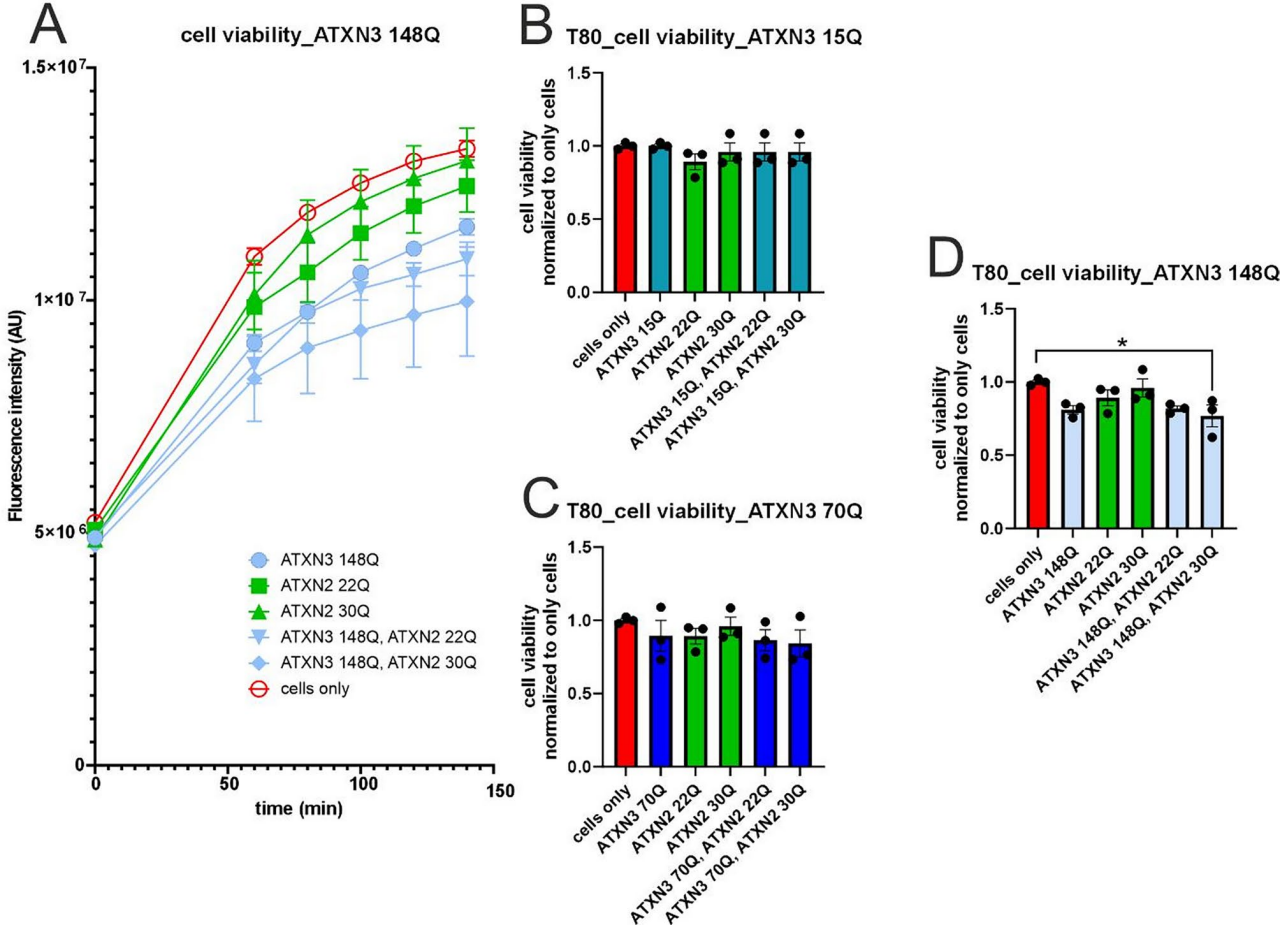
Cell viability decreased with expression of expanded repeat length in ATXN3, especially when co-expressed with intermediate ATXN2 30Q. **A**) Measurement of the fluorescence intensity signal (AU) over a period of 140 min with cells only, single-transfected ATXN3 148Q, ATXN2 22Q and ATXN2 30Q, as well as co-transfected ATXN3 148Q, ATXN2 22 and ATXN3 148Q, ATXN2 30Q. **B**-**D**) Measured intensity levels (*n* = 3, per construct) at time T = 80 min, normalized to cells only, analyzed with ordinary one-way ANOVA. B) Cells single-transfected with ATXN3 15Q, as well as single- and co-transfected with ATXN2 22Q or 30Q, respectively. **C**) Single-transfected cells with ATXN3 70Q, ATXN2 22Q or 30Q and respective co-transfected combinations. **D**) Single-transfected cells with ATXN3 148Q, as well as single- and co-transfected with ATXN2 22Q or 30Q. A significantly reduced cell viability was detected between the non-transfected cells (only cells) and cells co-transfected with ATXN3 148Q, ATXN2 30Q. Q = glutamine, * *p*<0.05

Finally, the cell viability test revealed that, over time non-transfected cells had the highest cell viability levels, followed by those of the ATXN2 22Q or ATXN3 30Q and ATXN3 15Q or ATXN3 70Q transfected cells; however, the co-transfected cells presented the lowest fluorescence intensity and therefore strongly reduced cell viability (Fig. 7B-C, Additional Fig. 6A-B). Additionally, these analyses revealed that the longer the ATXN3 repeat length was, the lower the fluorescence signal, and therefore, the lower the cell viability was (Fig. 7A, Additional Fig. 6A-B). Statistical analysis revealed a significant reduced cell viability in ATXN3 148Q with ATXN2 30Q compared to non-transfected cells (only cells) (ordinary one-way ANOVA, *p* = 0.0449) (Fig. 7A, D).

## Discussion

Our study represents a comprehensive analysis of various aspects of *ATXN2* and its impact on SCA3 pathogenesis, including cross-species evaluations of human blood and post-mortem human brain tissue, SCA3 mice and cell culture material. Overall, we analyzed genetic alterations in patient-derived material, including *ATXN2* with an intermediate CAG repeat, as well as a previously described 9bp duplication, and the expression of ATXN2 isoforms in a large European SCA3 cohort [34].

The occurrence of 22 CAG repeats in *ATXN2*, the most frequent variant was confirmed by our data [24–26, 44]. Focusing on the intermediate *ATXN2* repeat (27–33 CAG), one study identified 76 of 2408 individuals (3.16%) among healthy controls with an intermediate repeat [25]. Our findings suggest the same proportion among CNTRs since we identified 2 (1.94%) subjects harboring an intermediate *ATXN2* CAG repeat. Concerning SCA3 MC, a study by Tezenas du Montcel et al. (2014) reported the occurrence of intermediate *ATXN2* alleles in SCA3 MC, with a frequency between 0% in a Japanese cohort (*n* = 0), 2% in a French cohort (*n* = 1), 5% in an American cohort (*n* = 6) and 7% (*n* = 28) in a large European cohort [7]. In our cohort we identified 11 (4.35%) SCA3 MCs with an intermediate *ATXN2* allele. As previously published and shown by our results here, the frequency of identified intermediate *ATXN2* alleles was greater in SCA3 MCs than in healthy controls. Additionally, we investigated the sequence of the CAG repeat and found the same patterns of homo- and heterozygote CAA interruptions (8CAG-1CAA-4CAG-1CAA-8CAG), with most probands carrying one to three CAA interruptions (data not shown), as already reported in multiple other studies [20, 32, 44–46]. Furthermore, we were able to detect CAA interruption at position 19 of the CAG repeat in two probands with an intermediate repeat length of 27 (8CAG-CAA-4CAG-CAA-4CAG-CAA-8CAG), which was previously described in ALS patients with repeat lengths of 27 and 29 [47]. Furthermore, SCA3 MC with an intermediate *ATXN2* allele demonstrated a slightly shorter *ATXN3* repeat length compared to SCA3 MC with a shorter *ATXN2* repeat. On this basis, we would expect a later AAO, a lower SARA sum score and INAS count, and a lower progression rate. In contrast, we found a tendency toward an earlier AAO and no differences in SARA and INAS when comparing the SCA3 MC with different *ATXN2* CAG repeat lengths. Here, our results are inconsistent with the literature to findings that the intermediate *ATXN2* allele acts as a modulating factor of an earlier AAO [7, 31, 48]. Also Tezenas du Montcal et al., (2014) could not replicate the outcome of an earlier AAO in the EUOSCA cohort with their chosen replicate cohorts [7]. Similarly, Jardim et al. (2003) reported no evidence for a correlation between *ATXN2* repeats and AAO, although no intermediate repeat but only repeat lengths of 23 CAG’s were examined [49]. Raposo et al., (2015) presented data from SCA3 subjects from Azores and reported that 5% of their subjects carried an intermediate *ATXN2* CAG repeat, and they showed a tendency toward an earlier AAO in SCA3 MC with an intermediate *ATXN2* repeat [31]. Owing mainly due to the rarity of intermediate repeats and the associated small cohort size, it remains uncertain whether the observed tendency towards an earlier AAO is in fact robust [7, 50]. The influence of an intermediate ATXN2 repeat on the AAO in SCA3 could also not be clarified in our large, highly standardized European SCA3 cohort.

To our knowledge, our study is the first that analyzed the relationship between *ATXN2* genetic alterations and clinical symptom manifestation (SARA and INAS) and symptom severity in SCA3 patients in detail. SCA3 patients often present with an “ataxia plus” syndrome in which the development of ataxia is accompanied by non-ataxic symptoms [14]. In a Chinese SCA3 cohort, rigidity was found to be the most frequent extra-cerebellar sign, while sensory symptoms and chorea occurred rarely, and myoclonus was not found [51]. While evaluating non-ataxic symptoms, we identified a combination of symptoms that usually occur related to polyneuropathy, mainly sensory symptoms, and parkinsonian symptoms, especially rigidity, which was significantly more common among SCA3 MCs with an intermediate *ATXN2* CAG repeat compared to probands with an intermediate short, medium or short repeat. Overall, rigidity, chorea and myoclonus were rarely detected in our cohort, whereas sensory symptoms were found in most subjects. The SCA3 MC showed a different pattern of clinical non-ataxic symptoms depending on *ATXN2* repeat length. Therefore, our study extends the previously described clinical pattern of the Chinese cohort [51], but these differences could be due to the geographical differences between the cohorts. Jardim et al. (2023) described an association between a long *ATXN2* allele (the longest allele described carried 23 CAG repeats) and the presence of fasciculations [49]. Furthermore, they described SCA3 probands with 22 *ATXN2* repeats as having no or mild facial fasciculations and no other associations between repeat length and the severity of neurologic findings, such as gait and limb ataxia, pyramidal findings, dysarthria, dysphagia, external ophthalmoplegia, nystagmus, eyelid retraction, dystonia, and optic atrophy [49]. In our cohort, fasciculations were not common in SCA3 MCs with longer *ATXN2* repeat lengths.

Another genetic alteration of *ATXN2* that we analyzed more closely in this study was the 9bp dup previously described by Laffita et al. (2021) [32, 52]. In the cohort described by Laffita et al., (2021), they reported a correlation between the 9bp dup and a lower AAO in SCA3 [32]. However, only 1 out of 28 SCA3, and 4 out of 823 CNTR were found to harbor the 9bp dup [32]. These very small numbers limit the ability to draw firm conclusions. In contrast, we present the largest SCA3 cohort used to date to investigate this 9bp region. Our cohort represents a significantly larger number of SCA3 MCs, and among 239 subjects, 10 (4.18%) were identified with a 9bp dup. Laffita et al. (2021) identified 4 individuals (0.48%) with 9bp dup in a group of 823 CNTR. Our control group, on the other hand, included 98 subjects, of whom 2 (2.04%) healthy probands were found to harbor the 9bp dup. In contrast to previously published studies [32, 52], no familial relationships were present in the 9bp dup group in our cohort. Our study revealed a shorter *ATXN3* CAG repeat in the 9bp dup group. As expected, owing to the *ATXN3* repeat length, the SARA sum score and INAS sum count were lower and the AAO was later. Interestingly, the DD was significantly shorter in the 9bp dup group. This could be partially explained by the greater AAO since both groups were similar in terms of age. However, the CSDP and “DD vs. SARA” suggest a faster disease progression in probands with 9bp dup in our cohort. Thus, we could not confirm the previously reported influence of the 9bp dup on an earlier AAO [32, 52] but rather showed the opposite effect. Nevertheless, our study indicates that the 9bp dup could affect the disease progression rate. Laffita et al. (2021) further postulated that the combination of the 9bp dup and an intermediate *ATXN2* CAG repeat of 29 CAGs leads to a reduced AAO [32]. None of our 12 identified 9bp dup probands were found to carry both an intermediate *ATXN2* repeat and a 9bp dup. Additionally, Laffita et al., (2021) described a co-segregation with a known *ATXN2* polymorphism, rs7969300 [32]. Our 9bp dup cohort showed all combinations at the SNP position (combinations G/G, G/A and A/A), and we could not find any co-segregation with the SNP rs7969300 (data not shown).

After the genetic aspects at the DNA level were evaluated, the first comprehensive and detailed evaluation of *ATXN2* isoform expression in the brain and blood of SCA3 MCs was performed. We focused on different *ATXN2* isoform and splice variants by comparing RNA sequencing data from blood and cerebellar samples of CNTR and SCA3 MCs. Our RNA analysis of *ATXN2* revealed generally greater expression of *ATXN2* isoforms in cerebellar tissue than in blood and greater expression in SCA3 MCs than in CNTRs. Earlier studies demonstrated that *ATXN2* isoforms are highly expressed in brain tissue [21], fibroblasts and the cerebellar cortex but are expressed at lower levels in the cerebellum and liver [40]. The total expression of *ATXN2* in the Human Protein Atlas is reported to be 28.6 tpm in the cerebellum [53], and in the GTEX portal, *ATXN2* is expressed in the cerebellum at 33.1 tpm (*n* = 241) and in whole blood at 4.05 tpm (*n* = 755) [54]. Furthermore, the alternative splicing of *ATXN2* was described to be dependent on several regulating factors, such as quaking I [55]. According to Ensembl, the splice variant type II [33, 40, 42] could not be identified as a variant missing only exon 10 but was identified in isoforms lacking additional exons. Splice variant type I (full-length forms) was detected with a significantly lower expression rate in cerebellar tissue and significantly higher expression rate in blood compared to type II for CNTR and MC group comparisons. Another variant (type IV) described lacking exon 21 [41] was detected in both blood and brain, and there was no significant difference between CNTRs and SCA3 MCs. As previously stated by Affaitati et al., (2001), splice variant types I, II and IV are detectable in several human tissues, including the brain, spinal cord, cerebellum, heart and placenta, which might be due to various functions of these splice variants in specific tissues [40, 41]. The variant type VI, lacking exon 24 [40], corresponds to an inhomogeneous group formed by several shorter protein-coding isoforms without the CAG region, which in total were detected in both tissues with similar expression in CNTR and SCA3 MC. In line with others, we confirmed the expression of specific isoforms in specific tissues but described for the first time differences between SCA3 MC and CNTR, mostly for splice variant types I and II.

We also investigated *ATXN2* at the protein level in SCA3 cell models and a mouse model. While studying the interaction of ataxin-2 and polyQ-expanded ataxin-3, Nóbrega et al., (2015) reported reduced ataxin-2 levels with further reductions with disease progression [43]. In contrast, in our study, no reduction in ataxin-2 protein abundance was detected in SCA3 knock-in mice compared with wildtype mice. Determination of ATXN2 abundance over the whole life span in mice revealed a significant genotype-independent reduction in ataxin-2. Additionally, no ataxin-2 co-localization into ataxin-3-containing aggregates was observed in our study. The DD-AGE analysis performed using cell culture lysates revealed also no co-localization. This is in contrast to previous results by Nóbrega et al., (2015) [43]. They demonstrated altered subcellular localization of ATXN2 from the cytoplasm into the nucleus when cells expressed the disease-causing ataxin-3, whereas wild-type ataxin-2 remained in the cytoplasm [43]. The UniProt.org database states that ataxin-2 is expressed mainly in the cytoplasm [56]. The same applies to the findings published by van de Loo et al. (2008), according to which ataxin-2 is distributed throughout the cytoplasm but preferentially associated with rER/membrane-bound ribosomes [57]. This phenomenon was found to be independent of species and can be reproduced in non-neuronal cells, neuronal cells and neural tissue [57]. Furthermore, Uchihara et al., (2001) revealed the co-localization of ataxin-2 and ataxin-3 in neuronal intranuclear inclusions (NIIs), indicating that these proteins are incorporated into NIIs with expanded polyglutamine repeats as well as without pathological expansion of polyglutamine [58]. We observed cytoplasmic staining of ataxin-2 in the deep cerebellar nuclei (DCN), cerebellum (cere), pons and hippocampus (gyrus dentatus) of SCA3 mice. The same mice that were stained with ataxin-3 presented high amounts of ataxin-3-positive aggregates in the corresponding brain regions. Nevertheless, ataxin-2 did not form together with ataxin-3 aggregates in SCA3 ki mice. The abundance of ATXN2 and ATXN3 was further investigated via western blot analysis. Overexpressed ataxin-2 protein was observed to have a higher molecular weight than endogenous ataxin-2 protein, which matches findings reported by Lastres-Becker et al. (2019). However, this size difference is too large to be explained by alternative splicing alone [40]. We showed via western blot, DD-AGE and filter trap analyses that the overexpression of ataxin-2, especially with an intermediate repeat length (30Q), significantly reduced the expression and aggregation of full-length ataxin-3, as well as the expression of cleavage products. This findings matches the previously described relationship between ataxin-2 and ataxin-3 upon re-establishing ataxin-2 levels by Nóbrega et al., (2015) [43]. A study by Lessing and Bonini (2008), on the other hand, suggested that pathogenic human ataxin-3 toxicity is critically dependent on ataxin-2 activity [22]. While co-expressing ataxin-2 with a non-pathogenetic ataxin-3 had no effect, co-expressing ataxin-3 with a polyQ-expansion led to dramatically enhanced cell degeneration in the Drosophila model [22]. The up-regulation of ataxin-2 was found to drive the formation of inclusions by pathogenic ataxin-3 and, therefore, enhances the toxicity of mutated ataxin-3 [22]. A reduction in ataxin-2 activity was shown to reduce SCA3 degeneration, indicating that normal protein activity is of central importance [22]. Overall, we could not confirm increased cell viability in cells with polyQ-expanded ataxin-3 overexpressing ataxin-2. In particular, the decrease in cell viability in cells expressing pathological ataxin-3 (148Q) and ataxin-2 with an intermediate repeat length (30Q) does not match our results of reduced ataxin-3 protein levels and aggregates when co-expressed with ataxin-2 and does not fit the results of Nóbrega et al., (2015) [43]. Interestingly, these results are in line with what Lessing and Bonini (2008) postulated that up-regulated ataxin-2 has negative and toxic effects on SCA3 [22]. Finaly, more studies on the impact of ataxin-2 on SCA3 pathogenesis are needed to obtain a clearer picture of the impact of ataxin-2 on disease manifestation and progression and the underlying mechanisms.

In conclusion, we present a comprehensive cross-species analysis of *ATXN2* in SCA3 pathogenesis, including the clinical relevance of genetic variants, isoform expression in the human brain and blood, and protein interactions in cell culture and mouse experiments. Taking advantage of the large and deeply phenotyped ESMI cohort, we contribute to a more precise understanding of the influence of the intermediate CAG repeat of *ATXN2* on SCA3, especially disease progression and non-ataxic symptoms. Furthermore, in mouse and cell culture experiments, we investigated the influence of an intermediate *ATXN2* repeat on *ATXN3* protein levels and aggregation. As the 9bp duplication is very rare and not expressed at the RNA level in the brain or blood, its potential as a modulator of the course of SCA3 is very limited. Our study provides a comprehensive and unprecedented analysis of all *ATXN2* isoforms and splice variants in cerebellar tissue as well as blood from SCA3 MCs and CNTRs. We believe that splice variants of ATXN2 have potential for future research concerning the influence on SCA3 pathogenesis.

## Electronic supplementary material

Below is the link to the electronic supplementary material.

Supplementary Material 1

## Data Availability

The raw data that support the findings of this study are available upon reasonable request from the corresponding author. The data are not publicly available due to privacy or ethical restrictions.

## Declarations

### Research involving animals

All animal work was performed with approval from the Ethics Committee of the University of Tübingen, Germany (HG3/13).
