# Supplementary material for: Influence of ATXN2 intermediate CAG repeats, 9bp duplication and alternative splicing on SCA3 pathogenesis
Source: Acta Neuropathol Commun. 2025 Jul 19;13:157. doi: 10.1186/s40478-025-02074-0 (PMC12275423; doi:10.1186/s40478-025-02074-0)
Supplement: Supplementary file 1 — Supplementary Material 1 [file 40478_2025_2074_MOESM1_ESM.pdf]

Supplementary data to

Influence of *ATXN2* intermediate CAG repeats, 9bp duplication and alternative splicing on SCA3 pathogenesis

Marilena Lauerer[1,2], Jennifer Faber[3,4,5], Nicolas Casadei[1,6], Magda M Santana[7], Georg Auburger[8], Michaela Pogoda[1,6], Jakob Admard[1,6], Lea Kaupp[1,2], Patricia Laura Kos[1,2], Mafalda Raposo[9], Manuela Lima[10,11], Luis Pereira de Almeida[12,13,14], Hector Garcia-Moreno[15,16], Paola Giunti[15,16], Jeroen de Vries[17], Bart P. van de Warrenburg[18], Judith van Gaalen[18,19], Marcus Grobe-Einsler[3,4], Berkan Koyak[3,4], Kathrin Reetz[20,21], Friedrich Erdlenbruch[22], Heike Jacobi[23], Jon Infante[24,25], Holger Hengel[26], Ludger Schöls[26], ESMI study group, Thomas Klockgether[3], Olaf Rieß[1,2,6], Jeannette Hübener-Schmid[1,2]*,

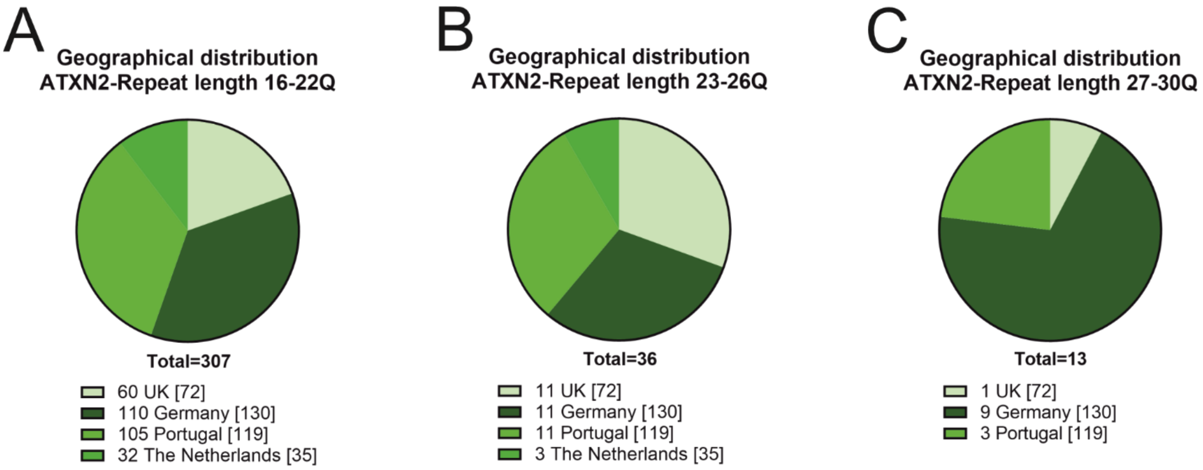

Additional Figure 1: Out of 356 ESMI participants (CNTR and SCA3 MC), 13 demonstrated an intermediate ATXN2 repeat (27-30Q) and most of them were identified in the German cohort. *A-C) Pie charts illustrate geographical distribution of the ESMI cohort divided into different groups of ATXN2 repeat length (16-22Q, 23-26Q and 27-30Q). Number in front of the country name corresponds to the total number of identified individuals from the respective country belonging to the respective repeat group, whereby the number in brackets corresponds to the total number of subjects from this country.*

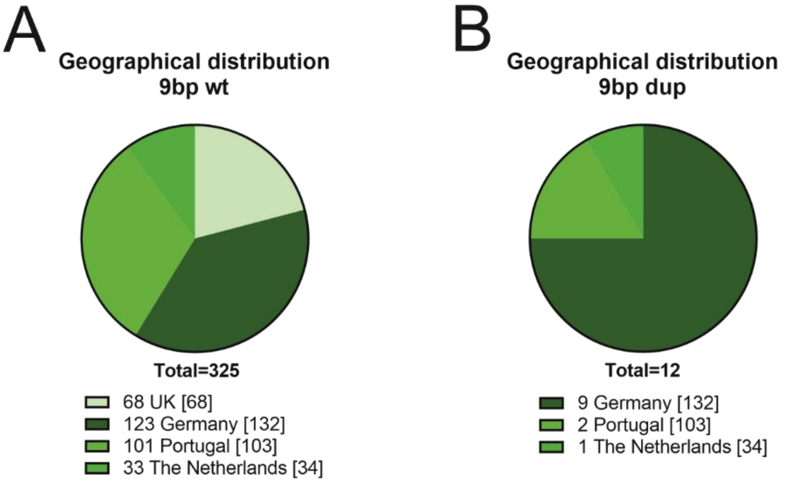

Additional Figure 2: Geographical distribution of ATXN2 9bp dup in the ESMI cohort showed that 75% of all identified ATXN2 9bp dup were found in the German cohort. *A) Pie chart demonstrating geographical distribution of ESMI probands which have no ATXN2 9bp duplication (9bp wt). B) Pie chart displaying geographical distribution of ESMI probands with an ATXN2 9bp dup. The number in front of the country name*

corresponds to the total number of identified individuals from the respective country belonging to the respective 9bp group, whereby the number in brackets corresponds to the total number of subjects from this country

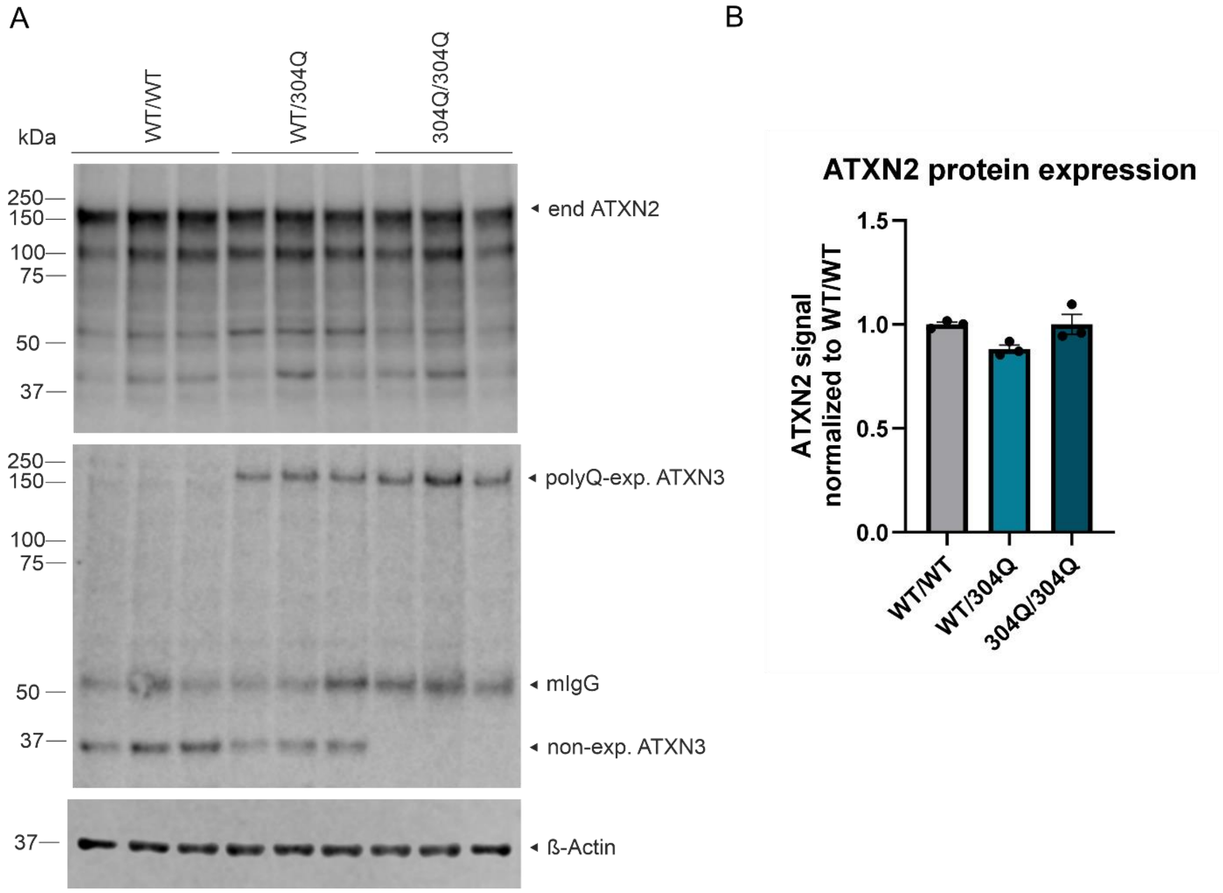

***Additional Figure 3*: Soluble ataxin-2 protein abundance is similar in SCA3 knock-in (ki) mice compared to wildtype.** *A) Heterozygous (WT/304Q) and homozygous (304Q/304Q) SCA3 ki mice compared to controls (WT/WT) were analyzed using western blot, to determine soluble ataxin-2 and ataxin-3 protein (genotype confirmation) abundance in whole brain lysates at the age of 3 months. B) Statistical quantification using Kruskal-Wallis test confirmed similar ataxin-2 abundance in all genotypes. n=3 per genotype, end ATXN2 = endogenous ataxin-2, polyQ-exp ATXN3 = polyQ-expanded ataxin-3, non-exp. ATXN3 = non-expanded ataxin-3, mIgG = mouse immunoglobulin. ß-actin was used as loading control.*

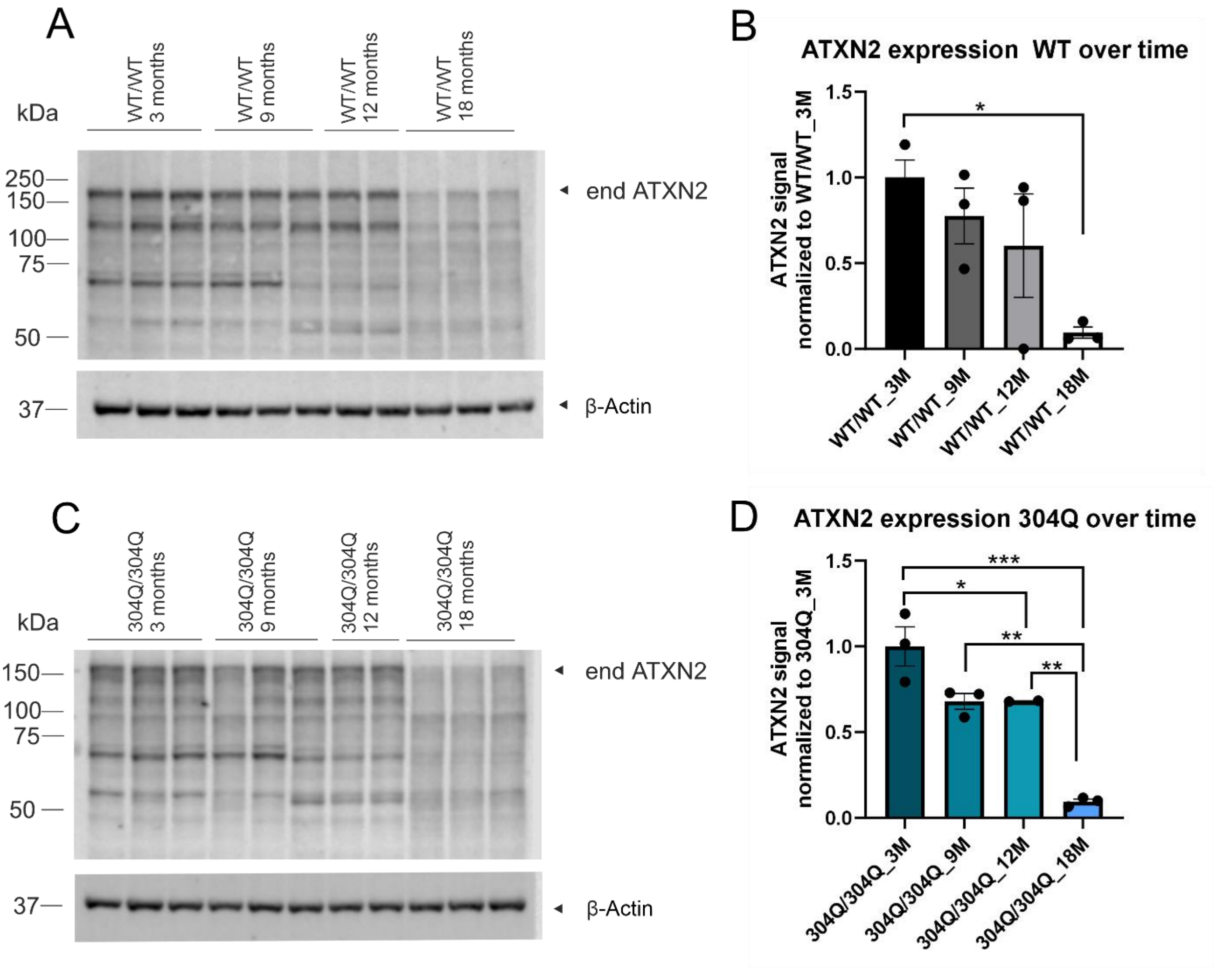

Additional Figure 4: Soluble ataxin-2 protein abundance is reduced with aging independent from genotype. *A) Western blot analyses of whole brain lysates of wildtype animals at 3, 9, 12 and 18 months. B) Statistical evaluation using Kruskal-Wallis test confirmed significant down-regulation with aging. C) Whole brain lysates of homozygous SCA3 ki mice (304Q/304Q) were analyzed using western blot immunostained with ataxin-2 specific antibody. D) The Kruskal-Wallis test revealed a significant down-regulation of ataxin-2 over time. n=3 per genotype, end ATXN2 = endogenous ataxin-2, 3M = months, 9M = 9 months, 12M = 12 months, 18M = 18 months. β-actin was used as loading control. * $p<0.05$, ** $p<0.01$, *** $p<0.001$*

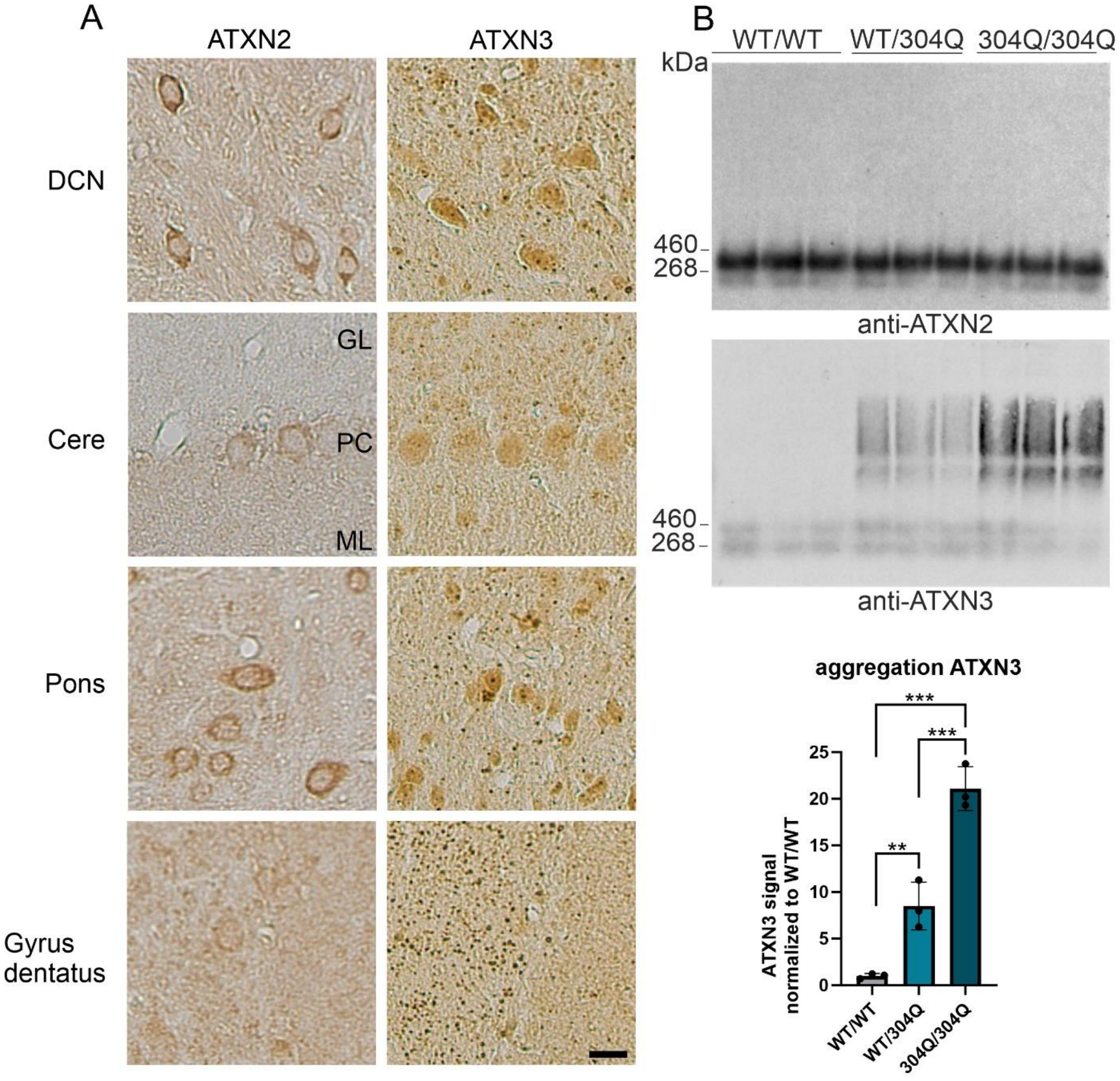

Additional Figure 5: Ataxin-2 is not part of ataxin-3 positive aggregates in SCA3 knock-in (ki) mice. *A) Immunohistochemistry (IHC) of 18-month-old homozygous SCA3 ki mice revealed cytoplasmic staining of ataxin-2 in the deep cerebellar nuclei (DCN), cerebellum (cere), pons and hippocampus (gyrus dentatus). The same mice stained with ataxin-3 revealed a high amount of ataxin-3 positive aggregates in the respective brain regions. N=3 per genotype and brain region. B) DD-AGE analyses confirmed IHC results and demonstrated no ataxin-2 positive aggregates in 18-month-old SCA3 ki mice. n=3 per genotype. Same blot was stained first for ataxin-2 and afterwards for ataxin-3. Kruskal-Wallis test confirmed highest ataxin-3 positive aggregate amount in 304Q/304Q ki mice. ** $p<0.01$, *** $p<0.001$*

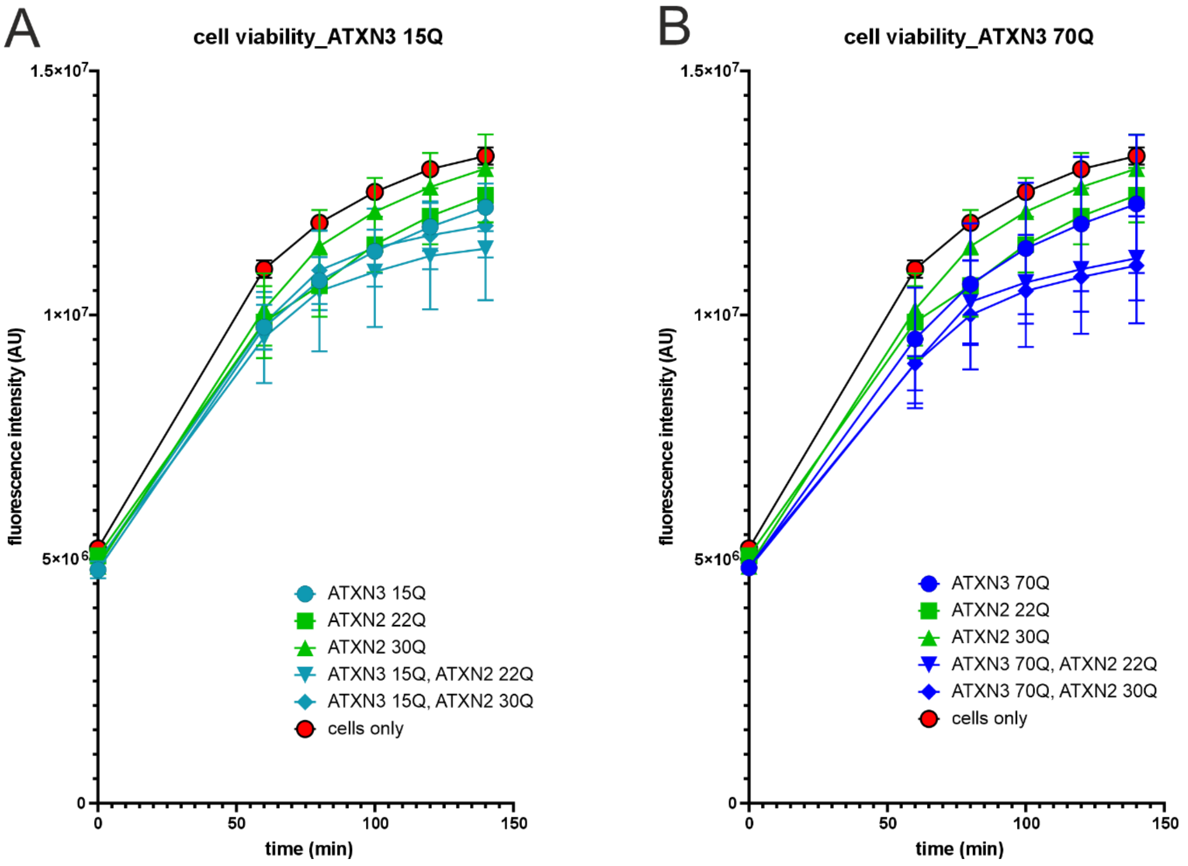

Additional Figure 6: Cell viability decreased with expression of expanded repeat length in ATXN3, especially when co-expressed with intermediate ATXN2 22Q and 30Q. *A-B) Measurement of the fluorescence intensity signal (AU) over a period of 140 min. A) Non-transfected cells were compared to single-transfected ATXN3 15Q, ATXN2 22Q and ATXN2 30Q, as well as co-transfected ATXN3 15Q, ATXN2 22Q and ATXN3 15Q, ATXN2 30Q. Fluorescence intensity levels do not differ strongly, but there is a tendency towards reduced cell viability of cells co-transfected with ATXN2, respectively. B) Single-transfected ATXN3 70Q, ATXN2 22Q and ATXN2 30Q, as well as co-transfected ATXN3 70Q, ATXN2 22Q and ATXN3 70Q, ATXN2 30Q were compared to non-transfected cells as positive control. Cell viability indicated reduced cell viability in co-transfected cells. Lowest viability was evaluated in ATXN3 70Q co-transfected with ATXN2 30Q. n=3 per construct, Q = glutamine*
